# Defining transcription factor nucleosome binding with Pioneer-seq

**DOI:** 10.1371/journal.pgen.1011813

**Published:** 2025-08-14

**Authors:** Maria Tsompana, Patrick D. Wilson, Vijaya Murugaiyan, Christopher R. Handelmann, Michael J. Buck

**Affiliations:** 1 Department of Biochemistry, Jacobs School of Medicine and Biomedical Sciences, State University of New York at Buffalo, Buffalo, New York, United States of America; 2 Department of Biomedical Informatics, Jacobs School of Medicine and Biomedical Sciences, State University of New York at Buffalo, Buffalo, New York, United States of America; Penn State University: The Pennsylvania State University, UNITED STATES OF AMERICA

## Abstract

Gene expression requires the targeting of transcription factors (TFs) to regulatory sequences often occluded within nucleosomes. To comprehensively examine TF nucleosome binding, we developed Pioneer-Seq. In Pioneer-seq a library of thousands of nucleosomes are formed from sequences containing a TF binding site (TFBS) variant in all possible nucleosome orientations and within the linker regions. Pioneer-seq has the unique ability to simultaneously examine nucleosomes created with various nucleosome positioning sequences and examine binding to *in vivo* targeted nucleosomes (ITNs). Pioneer-seq can be applied to address various mechanistic models for TF-nucleosome binding directly and can be used to uncover inherent TF-interaction differences. To demonstrate Pioneer-seq, we examined nucleosome binding by OCT4, SOX2, KLF4, and c-MYC. Our results demonstrate that all studied TFs can bind at nucleosome edges and nucleosome sequence is the primary factor regulating TF binding. In addition, KLF4 can bind to a non-canonical TFBS located 20 bp from the nucleosome dyad. Examination of ITNs showed binding differences between the TFs, with KLF4 and SOX2 binding more often near nucleosome centers. Overall, our results demonstrate differences in how TF recognizes their TFBS within a nucleosome and begins to define the mechanistic requirements for pioneer factor binding.

## Author summary

In human cells, DNA is packaged into chromatin, where it is wrapped around proteins called histones to form spool-like structures known as nucleosomes. This packaging helps organize the genome, but nucleosomes can make it harder for proteins called transcription factors to bind DNA and turn genes on or off. Some transcription factors, called pioneer factors, can bind DNA within nucleosomes, but the rules behind this ability remain unclear. To study this, we developed Pioneer-seq, a method that measures how strongly transcription factors bind thousands of different nucleosomes. Pioneer-seq tests each DNA binding site at every possible position within the nucleosome and includes both artificial sequences and real genomic sites where transcription factors and nucleosomes co-occur. We tested three pioneer factors—OCT4, KLF4, and SOX2—and one non-pioneer factor, MYC, and found that all four factors could bind at nucleosome edges, and that nucleosome DNA sequence played a key role in determining binding strength. We also discovered that KLF4 and SOX2 could access binding sites near the nucleosome center, depending on the context. Our results show that DNA sequence and nucleosome structure shape transcription-factor binding, and nucleosome binding is not unique to pioneer factors.

## Introduction

The interactions between proteins and chromosomal DNA underlie basic nuclear processes, such as transcriptional regulation, DNA replication, repair, and recombination, chromosome segregation, and epigenetic inheritance, as well as many fundamental biological responses, including cell growth, division, and differentiation, embryonic development, environmental stress responses, apoptosis, and disease state development. According to the human protein atlas, 8,887 proteins localize to the nucleus, with an estimated 1,500 DNA-binding transcription factors (TFs) [1]. Most TFs preferentially bind nucleosome-free DNA, which appears to be a conundrum during development because many gene regulatory regions are in nucleosomal DNA. However, a few TFs belong to a specific class, known as pioneer factors, that can bind to closed chromatin and open nucleosomal domains [2–6].

Pioneer factors cannot bind all their targets throughout the genome, indicating constraints to their binding abilities. There is evidence that the location of the TF binding site (TFBS) within a nucleosome (known as the translational setting) is one determinant of TF-binding abilities [6]. For example, binding of the glucocorticoid receptor at its TFBS near the nucleosome edge is 4-fold greater than at an identical site positioned 20 bp from the nucleosome dyad [7]. The translational setting can inhibit TF binding 2- to 100-fold [8–11]. TF binding is also influenced by the orientation of a TFBS on a nucleosome (known as the rotational setting) that results from the twist of the helical DNA structure. FoxA binds to its well-defined target site in the *Alb* enhancer (5–15 bp from the nucleosome dyad in liver cells) [12] only at specific rotational settings [13]. The ways by which TFs recognize their TFBSs (e.g., partial motif recognition) also likely influence their ability to bind nucleosomal DNA [14].

Until recently, studying TF binding to nucleosomal DNA relied on low-throughput assays in which nucleosomes were reconstituted *in vitro* on defined DNA templates known as nucleosome positioning sequences (NPSs), which are sequences that favor stable and reproducible nucleosome assembly at specific positions. In these assays, nucleosomes containing a single TFBS were incubated with increasing concentrations of a regulatory protein [15]. To examine different TFBSs or to determine their optimal translational or rotational settings within the nucleosome, each condition required a separate assay. More recently, methods like NCAP-SELEX and SeEN-seq have expanded the ability to study TF–nucleosome interactions at larger scales. NCAP-SELEX uses randomized DNA libraries that are reconstituted into nucleosomes and subjected to TF–nucleosome enrichment followed by high-throughput sequencing [16]. While this approach reveals general TF–nucleosome binding preferences, the randomized sequence context means that each TFBS is situated in a different nucleosome sequence, making it difficult to isolate the effects of nucleosome position from those of the surrounding DNA sequence. Other techniques, such as competitive nucleosome-binding assays and SeEN-seq, combine electrophoretic mobility shift assays (EMSAs) with defined nucleosome libraries and sequencing [4,5,17]. However, these methods are limited in scale and typically rely on a single nucleosome-positioning sequence, such as Widom 601, which limits the ability to test how TF binding changes depending on the nucleosome-positioning sequence.

To overcome these limitations, we developed Pioneer-seq, a high-throughput assay that maps TF binding at base-pair resolution across multiple NPSs. Unlike NCAP-SELEX, which embeds TFBSs in variable sequence contexts, Pioneer-seq shifts a defined TFBS across fixed nucleosome sequences, allowing the effects of translational and rotational settings to be measured directly. In contrast to SeEN-seq, which uses a single NPS (Widom 601), Pioneer-seq uses multiple NPSs, enabling comparisons across distinct nucleosome architectures. The library also includes *in vivo*–targeted nucleosomes (ITNs), enabling binding to be assessed in genomic regions where TFs and nucleosomes co-occur. Together, these features make Pioneer-seq uniquely suited for dissecting how TF binding is influenced by translational and rotational settings within the nucleosome.

## Description of the method

Pioneer-seq expands on preexisting competitive nucleosome-binding assays by examining thousands of nucleosomes, which include nucleosomes based on highly characterized NPSs and nucleosomes based on ITNs. Pioneer-seq uses 5S rDNA [18], MMTV LTR [19], and the artificial synthetic sequence Widom 601 DNA as NPSs [20]. The use of these highly characterized NPSs is advantageous due to the availability of their defined structures and dynamics [15]. To analyze each TF that will be studied, one of its TFBSs is incorporated into each of the three NPSs at intervals of 1 base pair. This includes sites within the core 147-bp nucleosome and outside in the linker regions. For each TF that will be studied, multiple TFBSs can be examined within a single experiment. Each NPS with the inserted TFBS is flanked by PCR primers (19–20 bp) for a total sequence length of 230 bp.

In addition to highly characterized NPSs, Pioneer-seq can also include genomic locations targeted *in vivo* by a TF of interest. These ITNs are defined by examining nucleosome positioning data from MNase-seq or NOMe-seq with TF binding data from ChIP-seq, ChIP-nexus, or Cut & Tag. The 191-bp sequences centered at the ITN are then filtered for predicted nucleosome formation ability [21] and the presence of the specific TFBS. In total, 7,500 sequences are designed for each library such that most sequences are not specific for any particular TF. All DNA sequences are synthesized as an Agilent 230-bp oligonucleotide library. Nucleosomes are then assembled with salt gradient dialysis using all nucleosome sequences simultaneously and free DNA is removed with a sucrose gradient.

**Fig 1:**
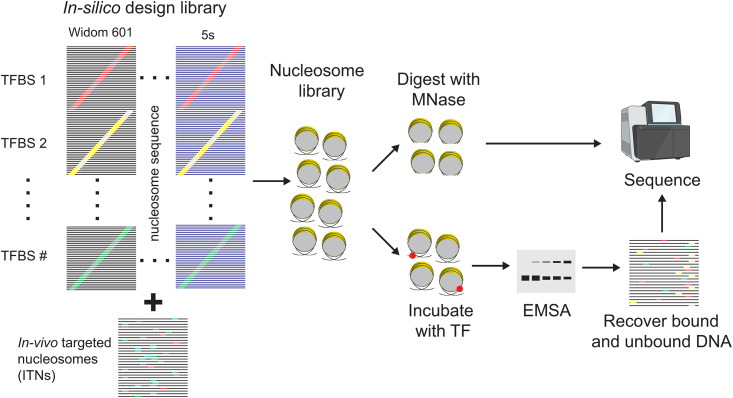
Overview of Pioneer-seq. With this method, 230-bp DNA sequences of interest are designed in batches of 7,500 sequences, including well-characterized nucleosome-positioning sequences (NPSs; Widom 601 or 5S rDNA) and sequences for *in vivo-*targeted nucleosomes (ITNs) (left). The method enables testing of binding-site variation and positioning, flanking-sequence content, and combinatorial binding events. Nucleosomes are formed and purified on all 7,500 sequences to generate a nucleosome library (middle). The entire nucleosome library is incubated with increasing amounts of a transcription factor (TF) of interest (bottom right). The TF-nucleosome complexes are separated by electrophoretic mobility shift assay (EMSA), and the bound and unbound DNA are recovered, quantified, and sequenced. Nucleosome positioning and accessibility for every DNA sequence in the nucleosome library are determined by digestion with micrococcal nuclease (MNase) (top right). The resulting DNA fragments are sequenced and mapped back to the initial library. TFBS, TF binding site. Figure partially created in BioRender.

The purified nucleosome library is used in binding assays in which the TF of interest is added at increasing concentrations. TF-nucleosome complexes are detected after a short incubation by separating the reaction mixtures on a native polyacrylamide gel (the first lane contains only nucleosomes to measure the background and input levels for each experimental replicate). Nucleosomes that the TF binds are identified by sequencing the DNA that is extracted and purified from shifted bands in the gel. The sequencing results are then analyzed and mapped to the original 7,500-nucleosome library. Fig 1 illustrates the Pioneer-seq workflow.

TF-nucleosome binding is quantified using the “relative supershift” metric, which compares the abundance of each nucleosome sequence in the shifted band to that of non-specific nucleosome sequences (i.e., those lacking a binding site for the transcription factor of interest) in the same gel lane [5]. This normalization controls for variability in gel loading, PCR amplification, sequencing, and potential binding to unintended motifs.

We determined nucleosome formation and positioning for the Pioneer-seq library using MNase-seq [22,23]. For these experiments, MNase-digested DNA from the nucleosome library is sequenced and mapped back to the 7,500-nucleosome library to determine nucleosome accessibility and positioning (S1–S3 Figs). For 601, the majority (90%) of nucleosomes appear to have a protected center within 10 bps of the 102 bp position. For 5S, there appear to be 3 populations of nucleosomes, one near the expected dyad position and two approximately 70 bp from the edges. MMTV nucleosome has a predominant nucleosome population (44%) near its expected dyad position with more variability than seen in the 601 nucleosomes.

## Verification and comparison

To evaluate Pioneer-seq, we examined nucleosome binding for the Yamanaka factors OCT4, SOX2, KLF4, and c-MYC [24]. OCT4, SOX2, and KLF4 have been shown to act as pioneer factors that directly bind to chromatin regions inaccessible to other TFs and subsequently trigger transcriptional competency by directing chromatin remodeling [25,26]. Despite their pioneering capabilities, OCT4, SOX2, and KLF4 cannot bind to all their TFBS within a genome [27].

To determine optimal TF concentrations for Pioneer-seq, we first tested each TF across a wide concentration range. At all concentrations tested, binding remained largely constrained to TFBSs located in linker DNA outside the nucleosome core (S4–S8 Figs).

**Fig 2:**
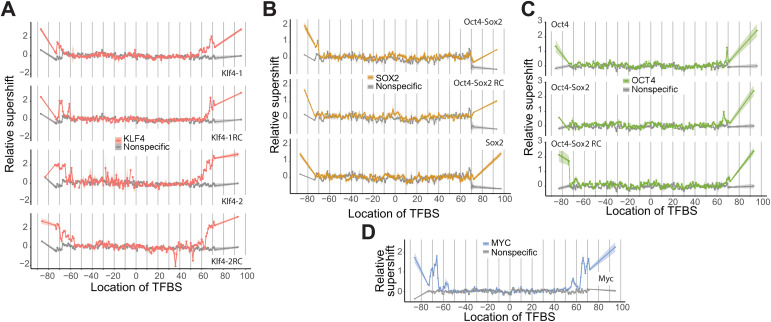
TF binding to the Widom 601 nucleosome. The specific and nonspecific TFBS is positioned across all possible locations along the 601 nucleosome with TFBSs in the left and right linkers to generate a total of 149 unique nucleosomes per TFBS. The relative supershift for each nucleosome is determined by counting the frequency of each sequence within the shifted band in the electrophoretic mobility shift assay and comparing it to that for nonspecific binding (i.e., binding to a sequence without the TFBS for that particular TF). This value is then normalized to the input ratio of nucleosomes (see Eq. 1). Shading around each line is SEM. Binding for KLF4 **(A)**, SOX2 **(B)**, OCT4 **(C)**, and MYC (D) is shown for nucleosomes with their specific TFBSs along with binding to a nonspecific TFBS nucleosome sequence (shown in gray). Breaks in the trace for Klf4-2 TFBS indicate missing data as a result of inefficient nucleosome formation. Klf4-1 is the canonical KLF4 motif (CCCCACCC) from JASPAR (MA0039.4), and Klf4-1RC is its reverse complement. Klf4-2 is a longer motif (GCCCCGCCCCGCCCC) derived from [29], and Klf4-2RC is its reverse complement. SOX2 refers to the monomer motif ACAATGG (JASPAR MA0143.3). OCT4 refers to the motif TATGCAAAT (JASPAR MA1115.1), and Oct4–Sox2 is a composite motif (CTTTGTTATGCAAAT) derived from known co-binding sites. MYC refers to the palindromic E-box motif ACCACGTGGT (JASPAR MA0059.1). RC, reverse complement.

Within nucleosomes, there is extensive binding variability depending on the NPS and TFBS. The Widom 601 NPS is the most extensively studied NPS and has been a model for studying nucleosome structures and dynamics [20]. In our experiments, 601 nucleosomes are the most efficiently formed nucleosomes (S9 Fig). The binding of OCT4, SOX2, KLF4, and MYC to the 601 nucleosomes was inhibited when their TFBSs were < 55 bp from the nucleosome dyad (Fig 2), similar to that observed for TP53 and TP63 in previous studies [4,5]. For KLF4, there were two TFBSs (with corresponding reverse complement sequences): Klf4–1 (CCCCACCC) is derived from the motif MA0039.4 from Jaspar [28], and Klf4–2 (GCCCCGCCCCGCCCC) is derived from the KLF4 long motif discovered in mouse embryonic stem cells [29]. KLF4 bound strongly to Klf4–1 only when the site was positioned >55 bp from the 601 dyad. Results for binding to Klf4–2 were noisier at some internal nucleosome positions because Klf4–2 appears to disrupt nucleosome formation. The results for the reverse complement sequences (Klf4–1RC and Klf4–2RC) are similar to those for the direct sequences. For OCT4, there is an OCT4 TFBS (TATGCAAAT) and a joint Oct4-Sox2 TFBS (CTTTGTTATGCAAAT). The binding of OCT4 to both TFBS is very similar, as the OCT4 target sequence is the same. For SOX2, there is a joint Oct4-Sox2 TFBS and a SOX2 TFBS (ACAATGG). SOX2 binds both sequences in the linkers and can bind the Oct4-Sox2 TFBS at the left nucleosome edge. The non-pioneer factor MYC was examined with a single palindromic TFBS (ACCACGTGGT) derived from the motif MA0059.1 from JASPAR [28]. MYC can bind its TFBS in the linker and when its TFBS is located > 55 bp from the dyad.

**Fig 3:**
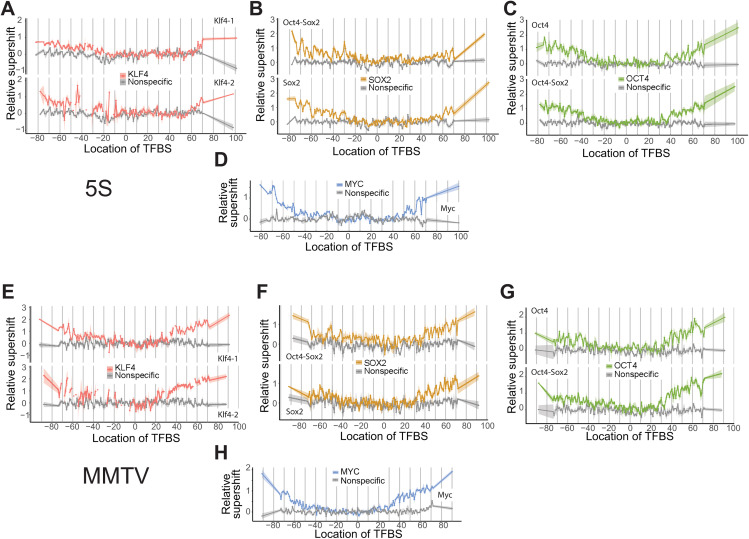
TF binding to 5S and MMTV nucleosomes. The TFBSs were positioned across all possible locations along the 5S (A–D) and MMTV (E–H) nucleosomes, with TFBSs in the left and right linkers. Relative supershifts for KLF4 **(A, E)**, SOX2 **(B, F)**, OCT4 **(C, G)**, and MYC (D, H) are shown for their specific TFBSs along with binding to a nonspecific TFBS nucleosome sequence (shown in gray). Breaks in the trace for Klf4-2 TFBS in panels a and e indicate missing data as a result of inefficient nucleosome formation.

Using Pioneer-seq, we can compare the binding of TFs across different NPS in a single assay. We compared binding to nucleosomes based on the NPSs of Widom 601, 5S rDNA, and the MMTV long terminal repeat. KLF4, OCT4, SOX2, and MYC were able to bind to their TFBSs closer to the dyad in 5S and MMTV nucleosomes than in 601 nucleosomes, with binding still inhibited within approximately ±30 bp from the dyad (Figs 2 and 3). These NPSs appear to allow transcription factors access to internal positions that are largely inaccessible in the 601 context. Binding was also asymmetric; there was increased binding on the left side of the 5S nucleosome and on the right side of the MMTV nucleosome. This asymmetry may reflect sequence-driven differences in nucleosome structure or unwrapping dynamics. We note that some of the increased internal binding in 5S and MMTV may also be influenced by the presence of alternative nucleosome conformations or positioning states in these libraries.

### Rotational and translational settings of nucleosome sequences drive binding differences

Because all 7,500 nucleosome sequences are exposed simultaneously to TFs in a single experiment, the binding to different NPSs or TFBSs can be directly compared. In general, we found that binding to TFBSs within the 601 nucleosome was strongly inhibited, whereas binding to TFBSs within the MMTV and 5S nucleosomes was less inhibited and increased as TFBSs approached the edges of the MMTV and 5S nucleosomes.

**Fig 4:**
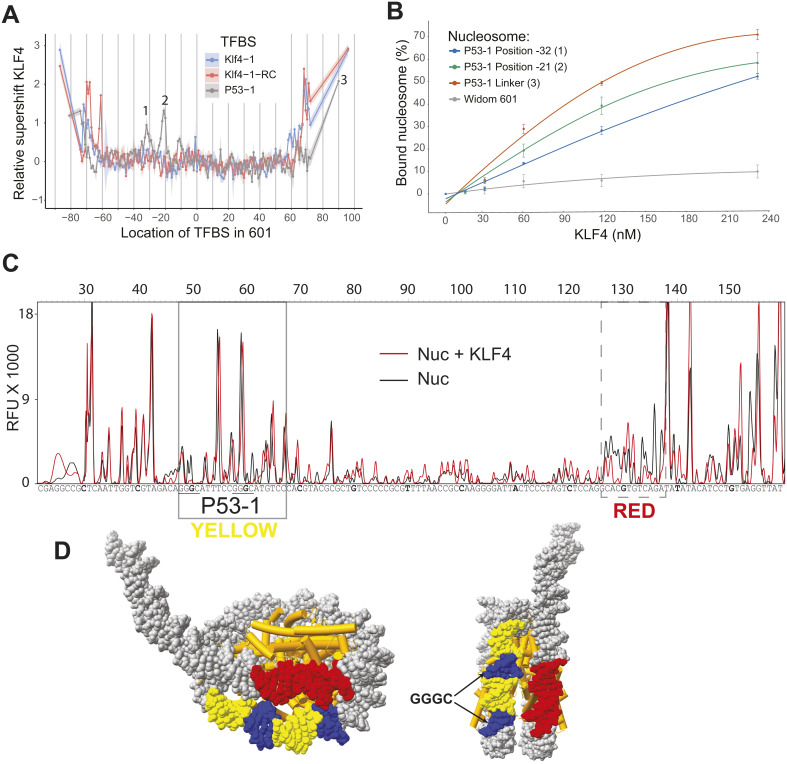
KLF4 binds an alternative TFBS. **(A)** Relative supershifts for KLF4 binding to Klf4-1 (CCCCACCC), Klf4-1RC (GGGTGGGG), and p53-1 (GGGCATGTCCGGGCATGTCC) within the 601 nucleosome; numbered locations indicate the nucleosomes validated by EMSA assays. RC, reverse complement. **(B)** Comparing the binding affinity for P53-1 containing nucleosomes at position -32 [1], at position -21 [2], in the linker [3], and to a control nucleosome. Bound nucleosome (%) was calculated via gel-shift assays featuring Cy5-labelled nucleosomes. **(C)** DNase-I footprinting of nucleosomes containing the p53-1 TFBS at position -21; an additional footprint observed on the neighboring gyre is indicated with the dashed-line box. Nuc, DNase-I digestion. **(D)** Model of the 601 nucleosome with the p53-1 motif highlighted in yellow, the two GGGC in blue, and the additional footprinted region in red.

In addition to the KLF4 TFBSs in the library, KLF4 can bind to the TFBSs for p53. At these sequences, KLF4 can bind at the linkers and at specific rotational and translational settings near the nucleosome dyad. Its preferred TFBS is unbound when positioned at the same internal locations. KLF4 binds the p53-1, p53-2, and p53-1RC TFBS in the linker regions but not as strong as the KLF4 TFBS (S8 Fig). On the other hand, when positioned near Super-Helix Location (SHL) -2 (-21 bp from dyad) within 601, the p53 motif (p53-1) and its reverse complement is bound (Figs 4A and S10). To confirm KLF4 binding to the p53-1 (GGGCATGTCCGGGCATGTCC) site on 601, we performed validation assays with EMSA and DNase-I footprinting. For EMSA validation assays we examined nucleosomes containing the p53-1 TFBS at two internal positions (-21 and -32) and in the linker region (Figs 4B, S11 and S12). For the DNase-I footprinting experiments, we examined the nucleosome containing the p53-1 TFBS at position -21 before and after KLF4 binding (Fig 4C). Changes in protection are distinguishable at the p53-1 TFBS. KLF4 appears to bind at two GGGC sequences spaced 10 bp apart in the p53-1 TFBS. The two GGGC sequences appear partially exposed in neighboring major grooves when that site is mapped onto the canonical 601 nucleosome structure (Fig 4D). The DNase-I footprinting also showed an additional footprint located at SHL 5.5. This region shown in red in Fig 4D is directly next to the p53-1 TFBS situated on the other DNA gyre, suggesting that KLF4 binding to p53-1 at SHL-2 disrupts the region near the entry/exit on the opposite side.

### Examining ITNs

In addition to well-characterized NPSs, ITNs at gene regulatory regions were included in the nucleosome library. These nucleosomes were identified using ChIP-seq binding data from the induction of OCT4, SOX2, KLF4, and MYC in fibroblasts during IPSC generation [27]. Nucleosome positions were defined by NOMe-seq from the fibroblast cell line (IMR90) [30]. After filtering nucleosomes by predicted nucleosome formation efficiency, 372 nucleosomes were designed. In total, ~ 20% of the ITNs failed to make stable nucleosomes for our assay. The remaining ITNs had a formation efficiency as good as or better than MMTV (S8 Fig and S1 Table).

**Fig 5:**
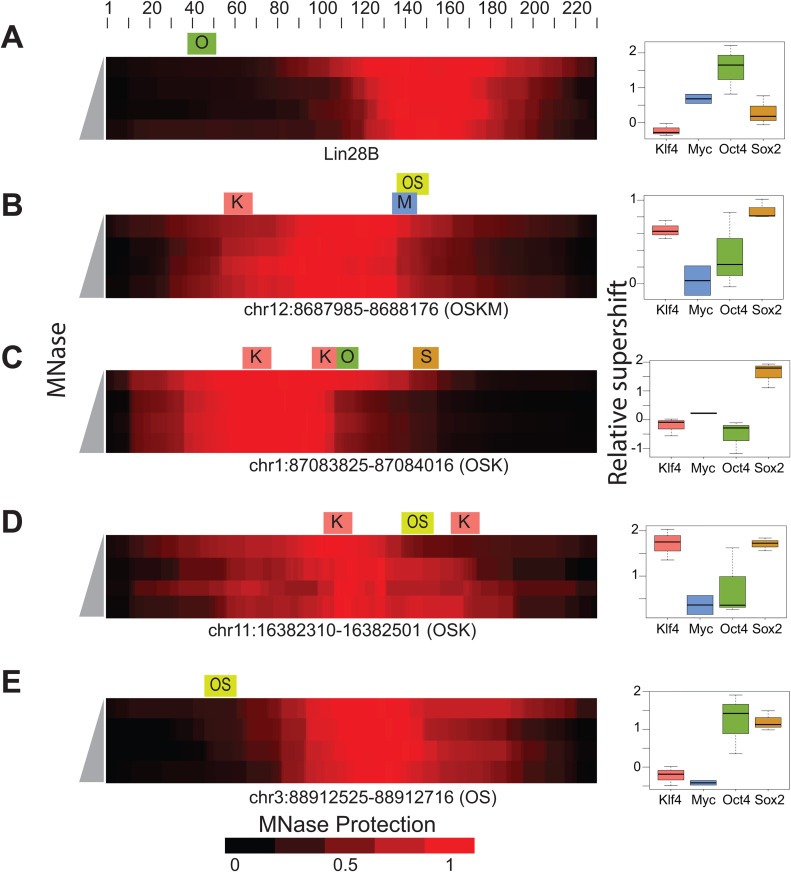
Binding to *in vivo*-targeted nucleosomes. MNase protection across each *in vivo*-targeted nucleosome (ITN) is shown as a heatmap (left), with shading from black to red indicating how strongly each base pair is protected from MNase digestion. Protection was measured by digesting the nucleosome library with MNase over a time course (0, 5, 10, 15, 20, or 25 min.; indicated in gray, left of each heatmap), then sequencing the resulting DNA. For each base pair, protection was calculated as the number of sequencing reads covering that base pair divided by the total number of reads mapped to that nucleosome. Darker red indicates bases that were more frequently protected from MNase digestion (i.e., more stably wrapped around histones), while black indicates regions that were more accessible. Colored boxes indicate transcription-factor binding sites: O – OCT4 (green), K – KLF4 (red), M – MYC (blue), S – SOX2 (orange), OS – OCT4/SOX2 (yellow). **(A)** Lin28B nucleosome sequence. **(B–E)** Four representative ITNs containing various combinations of TFBSs from the Yamanaka factors OCT4, SOX2, KLF4, and MYC. Box plots to the right of each heatmap show the relative supershift for each transcription factor on the corresponding nucleosome. In this figure, a single relative supershift value is reported per TF per ITN, representing the overall binding to that full nucleosome sequence.

The Lin28B nucleosome is an ITN that has been examined by multiple groups [14]. We defined the nucleosome-protected region for Lin28B with MNase-seq and showed a protected region centered at 150 bp with an OCT4 TFBS at position 38–46 bp (Fig 5A). Pioneer-seq results show that only OCT4 can bind specifically to the Lin28B nucleosome. Although prior in vitro studies also reported SOX2 binding to Lin28B, competition assays indicated that this binding was largely non-specific and readily competed by excess non-specific DNA [14]. The lack of SOX2 binding in Pioneer-seq likely reflects its competitive design, favoring the detection of strong, specific interactions and filtering out weak or non-specific ones. These results are consistent with DNase-footprinting and binding-specificity assays for KLF4, MYC, OCT4, and SOX2 [14]. The previously characterized ITNs located at ALBN1, NRCAM, CX3CR1, and ESRRB were also included within this library (S13 Fig). Other ITNs that were bound by OCT4, SOX2, KLF4, and MYC are shown in Fig 5B–5E.

**Fig 6:**
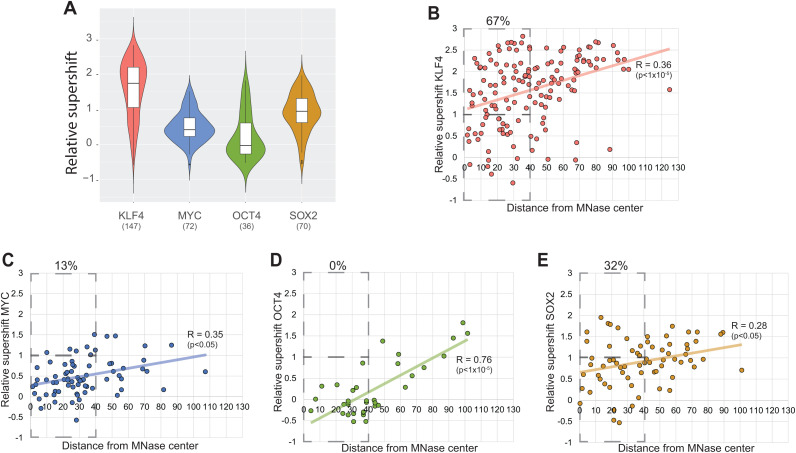
Binding to *in vivo-*targeted nucleosomes. **(A)** Violin plot showing relative supershifts for KLF4, MYC, OCT4, and SOX2 binding to *in vivo-*targeted nucleosomes (ITNs) containing their transcription factor binding sites (TFBSs). **(B-E)**, Relative supershifts for binding to ITNs compared to the distance of the TFBS distance from the center of MNase protection.

By examining the Oct4-Sox2 sites, we can directly compare OCT4 and SOX2 binding at their TFBSs at the same nucleosome positions. At most Oct4-Sox2 TFBSs, SOX2 can bind, while OCT4 only binds when the TFBS is outside the protected nucleosomal region (Figs 5B, 5D, 5E and S14). To compare binding between factors at ITNs, we have plotted the relative supershift for all ITNs containing a binding site. KLF4 and SOX2 bind to a larger percentage of their ITNs than MYC and OCT4 (Fig 6A).

To understand the role of nucleosome positioning for the ITNs, we mapped the TFBS in relationship to the center of MNase protection (Fig 6B–6E). The binding of KLF4, OCT4, SOX2, and MYC are significantly correlated with the distance of their TFBSs from MNase-protection centers. To examine binding near the nucleosome center, we selected the region 40 bp from the MNase center and determined the percentage of bound sites to the total number of sites within 40 bp. KLF4 bound 67%, SOX2 32%, MYC 13%, and OCT4 0%. This suggests that KLF4 and SOX2 may have a special ability to target some of their TFBSs within a nucleosome.

## Applications

Pioneer-seq resolves TF-nucleosome interactions at base-pair resolution using a custom library of nucleosomes assembled on well-characterized positioning sequences and on genomic loci known to form nucleosomes. By systematically shifting TF binding sites across defined positioning sequences, the assay directly tests how translational and rotational setting, as well as nucleosome sequence, influence TF binding. The library can also include genomic sequences where TFs and nucleosomes co-occur *in vivo* to examine TF binding in a biologically relevant genomic contexts. Because the same nucleosome library can be tested separately with different TFs, Pioneer-seq allows direct comparisons of TF-specific nucleosome-binding preferences.

Pioneer-seq can also be adapted to probe the structural determinants of TF-nucleosome binding more deeply. Mutant or truncated TFs can be assayed to pinpoint residues or domains required for nucleosome engagement or cooperative binding. Likewise, nucleosomes assembled with histone variants (e.g., H2A.Z or H3.3) or specific post-translational modifications (e.g., H3K27ac) can reveal how chromatin features alter nucleosome stability and TF binding. By assaying two TFs together, Pioneer-seq can also be adapted to test cooperative engagement at shared nucleosomal sites; binding-site architecture can then be systematically varied to determine how translational setting, site spacing, and rotational setting impact TF co-occupancy. This broad range of capabilities makes Pioneer-seq a powerful tool for defining how DNA sequence, nucleosome structure, and TF cooperativity collectively shape TF-chromatin binding.

## Discussion

Pioneer factors are a proposed class of TFs that can bind inaccessible genomic regions and then facilitate the binding of other TFs. This ability is attributed to specific DNA-binding domains that can target their binding sites within a nucleosome [31] and bind to partial motifs that are accessible on the nucleosome surface [14]. However, recent studies with the archetypal pioneer factor FOXA1 and its non-pioneer cofactor HNF4A have shown that binding is defined by the TFBS, specifically its density and affinity, rather than differences in TFs themselves [32]. Our results suggest a more complex model.

The TFs we tested bound most strongly at the nucleosome edge, but this differed according to the NPS being bound. These results are consistent with a dynamic partial unwrapping of DNA from histones at sites where the DNA enters or exits the nucleosome, exposing the DNA to TFs [33,34]. Edge binding would heavily depend on TFBS affinity and the number of sites. We also found that KLF4 targeted sites close to the nucleosome dyad at specific rotational settings, with an alternative TFBS (p53).

Our data newly reveal that KLF4 can directly bind to p53 binding sites within nucleosomes, specifically when the site is positioned near SHL-2 in the Widom 601 sequence. Although previous studies have suggested that KLF4 and p53 can cooperatively regulate gene expression [35–38], direct binding of KLF4 to p53 binding sites had not been reported. We did not observe this interaction in other NPSs, and it may reflect sequence- or structure-specific compatibility at that position. KLF4 appears to be binding to two partial motifs (CCCG) located in consecutive partially exposed major grooves, similar to the nucleosome binding of another zinc-finger TF, GATA3. GATA3 binds to split 5′-GAT-3′ motifs in solvent-exposed major grooves [39]. KLF4 will only bind the non-typical motif at these internal locations, which suggests that the partial motif or neighboring bases provide a structural binding context that is missing for other TFBSs. Our observation that KLF4 binds internal p53 sites in 601 but not in other NPSs suggests that nucleosome structure also facilitates certain binding events. Although the overall nucleosome structure is consistent across different NPS, certain patterns of dinucleotides cause differences in the groove width and helical deformation of nucleosomal DNA [40,41]. Indeed, groove width and the extent of helical deformation vary substantially among different sites with the same rotational settings [42,43]. Single-molecule DNA-unzipping experiments have shown that position-specific histone-DNA interactions also vary across the nucleosome [44]. These findings may have biological significance, since KLF4 and the p53-family member p63 are known to coordinate gene regulation during skin development and jointly target super-enhancers [45].

Our results for SOX2 and OCT4 for the Widom 601 NPS are similar to the previous shown by SeEN-seq [17]. Michael et al. (2021) showed binding of OCT4 only at the nucleosome edge, consistent with our results [6]. For SOX2 they used only the DNA binding domain and did not see any significant binding within 65 bp of the dyad. In our experiments, we used full-length SOX2 and identified binding only at the very edge -70 bp, which was not included in their experiments. For Lin28B, a recent study attempted to define cryo-EM structures for OCT4 and SOX2 binding at the Lin28B nucleosome [46]. Their results for OCT4 showed binding only at the OCT4 TFBS located within the linker region. They were unable to observe SOX2 binding, suggesting that it doesn’t stably bind to this nucleosome.

One of our more striking findings was that Myc-Max exhibited detectable binding to nucleosomal DNA (Figs 1 and 2), despite being widely considered a non-pioneer factor [47–49]. This suggests that the sheer ability to engage nucleosomal DNA does not qualify a transcription factor as a pioneer. Rather, pioneer factors appear distinguished by their ability to initiate chromatin remodeling, often through the recruitment of ATP-dependent chromatin remodelers (e.g., SWI/SNF complexes) or histone-modifying enzymes (e.g., p300/CBP) [50–52], an ability Myc-Max likely lacks. To elaborate by way of example: The canonical pioneer factor Oct4 directly interacts with SWI/SNF components like BRG1 to support chromatin engagement and increase accessibility at previously closed regulatory elements in embryonic stem cells [53,54], thereby enabling it to function as a pioneer factor. In contrast, Myc tends to operate within already accessible chromatin and does not recruit BRG1 or related remodeling complexes [47,55], and thus lacks the chromatin-opening capability that defines pioneer-factor activity.

The Widom 601 sequence is a synthetic construct derived from a SELEX experiment designed to isolate DNA sequences with high histone-octamer affinity [56]. While valuable for their tight positioning and experimental reproducibility, Widom 601 nucleosomes are far more stable than most nucleosomes *in vivo* [57–59]. Their use as NPSs in TF binding assays *in vitro* may thus limit detection of binding events that rely on transient unwrapping typical of nucleosomes *in vivo* [46,60,61]. Still, this hyperstability makes Widom 601 an ideal template for high-throughput assays like Pioneer-seq, where consistent nucleosome positioning is essential for dissecting how each translational and rotational setting impacts TF-nucleosome binding. To complement this highly stable, synthetic template, we also incorporated two additional templates into the Pioneer-seq nucleosome library, derived from naturally occurring NPSs: the 5S rDNA and MMTV promoter sequences. 5S and MMTV, like Widom 601, are well-characterized NPSs that form tightly positioned nucleosomes, but they are less intrinsically stable [57]. By incorporating these three NPSs as templates, Pioneer-seq captures a broader range of sequence-encoded nucleosome dynamics and thus a broader range of TF-nucleosome binding behaviors.

There are still many questions about how TFs can bind nucleosomal DNA that can be addressed with Pioneer-seq. The vast majority of studies on nucleosomes have used the Widom-601 NPS. This sequence is extremely well-studied and is the sequence used for 114 structural studies. Widom 601 forms nucleosomes *in vitro* very efficiently in a single predominate position, allowing reproducible and well-defined structures [15]. 5S and MMTV nucleosomes are biologically derived sequences that have been used for various studies [40,62]. 5S and MMTV nucleosomes are not as stable as 601 and form with reduced efficiency and can have multiple positions (S3 and S9 Figs). In this study, the nucleosome-binding abilities of each TF depended on the NPS being bound. In general, 601 nucleosomes were strongly inhibitory for TF binding, whereas 5S and MMTV nucleosomes were only inhibitory when the TFBSs were close to the nucleosome dyad. These results suggest that TF binding may be best understood by examining ITNs and model nucleosomes. The use of ITNs does have limitations because the exact positioning of the dyad is unknown, and weak nucleosome formation could limit specific sequences from being tested. For Pioneer-seq, we propose using ITNs along with a model nucleosome in which the TFBS can be positioned in all possible nucleosomal locations.

The ability of some TFs to bind a specific nucleosome site appears to depend on the binding sequence. This suggests that specific sites that require a pioneer factor may also have an alternative motif. The bases flanking the motif could impact nucleosome binding by affecting the structural presentation of the TFBS. For the TFs tested here, we only examined a few TFBS sequences and have not exhaustively characterized these TFs. Alternative sequences, such as the P53-1 TFBS for KLF4, was only discovered by happenstance. TF binding could also be affected by events that influence the shape of the DNA around the nucleosome, such as DNA methylation, histone variants, and histone modifications. In the future, Pioneer-seq can be used to investigate these possibilities.

Lastly, transcription is regulated by a complex of multiple TFs that bind proximal regulatory regions. These multi-TF binding events can be directly cooperative, as seen when TFs physically interact, or indirectly cooperative to displace the nucleosome [63]. Pioneer-seq is ideally suited to testing various models of cooperativity between TFs and enabling the mechanistic dissection of these crucial regulatory events. We are currently developing dual-factor Pioneer-seq experiments to directly investigate cooperative binding between factors in a nucleosomal context.

In summary, Pioneer-seq is a powerful method for investigating the essential first step in gene regulation, the binding of TFs to inaccessible DNA located within nucleosomes. Due to its nature of comparing specific binding to non-specific binding across a whole nucleosome library, Pioneer-seq allows the direct comparison of sites located in various nucleosome positions, with differing NPSs, and with varying TFBSs. Pioneer-seq can be applied to address various mechanistic models for TF-nucleosome binding directly and can be used to uncover inherent TF-interaction differences.

## Materials and methods

### Pioneer-seq library design

Three nucleosome positioning sequences (NPS), namely Widom 601, 5S rDNA, and mouse mammary tumor virus (MMTV)-A, were used to form stable nucleosomes. Each sequence has been characterized by multiple biochemical assays [64] and was scanned for the presence of binding sites for the transcription factors (TFs) of interest using FIMO; sequences were modified to remove the binding sites [65]. Sequences were generated with the TF binding site (TFBS) of interest placed at every base pair position in the nucleosome, with sites in both linker regions. In total, 149 sequences were designed for every TFBS with each NPS.

*In vivo*-targeted nucleosomes (ITNs) were determined by integrating datasets from chromatin immunoprecipitation with sequencing (ChIP-seq) and nucleosome positioning datasets. Locations bound by OCT4, SOX2, KLF4, or MYC were determined from ChIP-seq datasets [27]. Bound sites were checked for the specific TFBS, and locations lacking an identifiable TFBS were removed. Nucleosome positions were determined from NOMe-seq (nucleosome occupancy and methylome sequencing) from IMR90 cells (GSM543311) using the DANPOS algorithm [66,30]. The position serving as the nucleosome center was then expanded to 191 bp and evaluated with a nucleosome scoring function [21]. The probability that the center base is part of a nucleosome was used as the probability score for each nucleosome. Nucleosomes with a score of <0.7 were removed from the design.

### Nucleosome library assembly

All nucleosome sequences were flanked by primer sequences to generate 230-bp sequences. The nucleosome library containing a total of 7,500 unique sequences was acquired from Agilent as a custom oligonucleotide library, which was amplified using Herculase II Fusion DNA polymerase in 100-µl reaction mixtures (1x Herculase II reaction buffer, 1 mM dNTPs, 200 pM Agilent library, 250 nM forward and reverse primers) with 15 PCR cycles. For a typical experiment, the DNA obtained from 11 reactions was purified with a QIAquick PCR purification kit (cat. no. 28104; Qiagen) and quantified with a NanoDrop spectrometer; fragment size was confirmed with a 2% agarose gel. Nucleosomes were then generated from H2A/H2B dimers and H3.1/H4 tetramers (NEB) by incubating the DNA sequences and the histones at an octamer/DNA molar ratio of 1:1.2 (in a solution containing 10 mM dithiothreitol [DTT] and 1.8 M NaCl) for 30 min at room temperature. The reaction mixture was transferred to a Slide-A-Lyzer MINI dialysis unit (10,000 MWCO, cat. no. 69750; Thermo Scientific). Dialysis was performed with 1.2 ml of the dialysis buffers at 4°C in 1.0 M NaCl for 2 h, 0.8 M NaCl for 2 h, 0.6 NaCl for 2 h, and TE buffer (pH 8.0) overnight at 4°C. Nucleosomes were then transferred to a clean 1.5-ml tube pretreated with 0.3 mg/ml bovine serum albumin (BSA). Nucleosome formation was then confirmed by 4% native polyacrylamide gel electrophoresis. Free DNA was removed from nucleosomes by using a 7%–20% sucrose gradient, and nucleosomes were concentrated and quantified via qPCR [4,5]. Nucleosomes were then stored at 4 °C for up to 1 month.

### DNA binding assay followed by EMSA

The protein-nucleosome binding assays were carried out by incubating the purified nucleosome libraries described above and human full-length KLF4 (Origene TP306691), OCT4 (Origene TP311998), SOX2 (Origene TP300757), and MYC (Origene TP301611) with MAX (Origene TP306812) (in 7 µl DNA binding buffer (10 mM Tris-Cl [pH7.5], 50 mM NaCl, 1 mM DTT, 0.25 mg/ml BSA, 2 mM MgCl_2_, 0.025% Nonidet P-40, and 5% glycerol) for 10 min on ice and then 30 min at room temperature. Protein purity was confirmed by Coomassie staining, and binding activity was validated by EMSA using their respective binding sites on naked DNA. Increasing concentrations of TF (0–456 nM) were added to 28 nM purified nucleosomes. Protein binding was detected by electrophoretic mobility shift assays (EMSAs) on 4% (w/v) native polyacrylamide gels (acrylamide/bisacrylamide, 29:1 [w/w], 7 × 10 cm) in 0.5 × Tris-borate-EDTA buffer at 100 V at 4 °C. Initial EMSA experiments are done across a wide range of TF concentrations to determine the optimal TF amount and to ensure the supershift is observed on the gel.

### DNA isolation and purification

After electrophoresis, DNA was imaged by staining with SYBR green (LONZA). All visual bands, as well as the bands at the same locations in the other lanes were excised from the gel. The chopped gel slices were soaked in diffusion buffer (0.5 M ammonium acetate, 10 mM magnesium acetate, 1 mM EDTA [pH 8.0], 0.1% SDS) and incubated at 50 °C overnight. The supernatant was collected, residual polyacrylamide was removed with glass wool, and the DNA was purified with QIAquick spin columns (Qiagen). The DNA concentration for each sample was determined by qPCR using a standard curve generated from a control sequence.

### Library construction and sequencing

Illumina sequencing libraries were generated using a two-step PCR method, with 8–12 amplification cycles for the first step, including four sets of primers designed to offset sequence reads and dephase the libraries during Illumina sequencing. The number of cycles for the first-round PCR was determined using the sample concentration determined by qPCR. Each sample was then indexed using Nextera dual indices (Nextera XT index primer 1 [N7xx] and Nextera XT index primer 2 [S5xx]). After each PCR, reaction mixtures were cleaned up with AMPure XP beads (Beckman Coulter). The concentration of each sample was determined using the Invitrogen Quant-iT dsDNA assay kit, and equal amounts of each sample DNA were pooled and sequenced on an Illumina NextSeq 2x150. Sequencing and quality control were performed at the University at Buffalo Genomics and Bioinformatics Core.

### Pioneer-seq analysis

Illumina sequence reads were processed with an automated Snakemake pipeline of applications to refine and identify the sequences present in the sample pool [67]. The 3′ ends of Illumina FASTQ reads with low-quality scores were removed with Cutadapt using a quality cutoff of 30 (-q 30) [68]. Forward and reverse FASTQC reads were merged with Vsearch (--fastq_mergepairs) only if they shared at least 20 overlapping nucleotides (--fastq_minovlen 20) and had no more than two mismatched nucleotides between them (--fastq_maxdiffs 2) [69]. Primer sequences present at the ends of FASTQ reads were removed with Cutadapt. FASTQ reads of >220 nucleotides (nt) or <174 nt were filtered out with Cutadapt (--maximum-length 220 --minimum-length 174) [68]. FASTQ reads were converted to FASTA format using the FASTX-Toolkit FASTQ-to-FASTA command [70]. FASTA reads were mapped to a sequence in the reference library of 7,500 nucleosome sequences with Vsearch (--dbmatched) only if they had alignment lengths of at least 150 nt (--mincols 150), had at least 98.5% similarity (--id 0.985), and were the query and database sequence pairing with the highest percentage of identity (--top_hits_only) [69]. The results were then analyzed relative to control/nonspecific binding (relative supershift).

To quantify TF–nucleosome binding while controlling for technical variability (e.g., gel excision, PCR amplification, library construction, and sequencing), we calculated a “relative supershift” value for each nucleosome sequence. This value compares binding of a specific nucleosome sequence to background levels observed for non-specific sequences lacking a binding site for the TF of interest.


$$\text{celative}\;\text{supershift}=\;{\text{log}}_{2}\left(\frac{\text{reads}\;\text{supershift}\text{N}}{\overset{―}{(\text{reads}\;\text{supershift}\text{NS})}}/\;\;\;\;\frac{\text{reads}\;\text{nucleosome}\;\text{band}\;\text{TF}\;\text{null}\text{N}}{\overset{―}{(\text{reads}\;\text{nucleosome}\;\text{band}\;\text{TF}\;\text{null}\text{NS})}}\right)$$ (1)

where N is one of the 7,500 nucleosome sequences, NS is the control nucleosome sequences, “reads supershift” is the supershift band, and “reads nucleosome band TF null” is the nucleosome band in the TF-null lane. The control non-specific nucleosome sequences are selected from the same nucleosome-positioning sequence (NPS) as the test sequence (e.g., 601, 5S, or MMTV) and contain binding sites for transcription factors other than the TF of interest. This ensures that comparisons are made between sequences with the same positioning properties but lacking a relevant binding site for the TF being tested. Non-specific TFBSs are checked for potential binding by the transcription factor of interest when located in the linker region of the nucleosome, where binding would be most likely to occur. For each transcription factor, over 500 control sequences are based on the 601 positioning sequence, and 298 are based on the 5S and MMTV positioining sequences.

Pioneer-seq was performed with multiple concentrations of TFs because of differences in inherent binding affinities and protein purity. An initial analysis of linker binding events was used to (i) confirm the specific binding of the TF of interest and (ii) define the TF concentration with the most significant binding signal compared to nonspecific binding. The relative supershift for a single TF concentration is presented throughout this manuscript: 57 nM for KLF4, 28 nM for OCT4 and SOX2, and 114 nM for MYC/MAX. Pioneer-seq was replicated 3 times for KLF4, OCT4, and SOX2 and was replicated twice for MYC/MAX. Every Pioneer-seq EMSA is shown in S4–S7 Figs.

### MNase-seq on nucleosome library

Nucleosome positioning for each sequencing in the library was determined with MNase-seq as previously described (S1, S2 Figs) [71]. The nucleosome library (0.2 pmol/μl) was digested by MNase (0.05 U/μl) in nuclease digestion buffer (10 mM Tris-HCL [pH 8.0], 2 mM CaCl_2_) over a time course (0–25 min) at 37 °C; digestion was stopped with 2% SDS and 40 mM EDTA). Each sample was then incubated with proteinase K (16 μg) for 1 h at 55 °C. The DNA was purified from the reaction and concentrated with the QIAquick PCR purification kit. The concentration of each sample were was determined with the Invitrogen Quant-iT dsDNA assay kit and equalized. Illumina sequencing libraries were generated using an NEBNext Ultra II DNA library prep kit. Individual samples were multiplexed and sequenced via Illumina MiSeq 2x150.

MNase-seq results were quality filtered (*q* > 30) and adapter trimmed using Cutadapt [72]. The quality reads were merged and mapped to the 7,500 nucleosome library sequences using Vsearch [69]. The read counts and end positions were used to measure MNase protection, which was calculated for each base pair as the ratio of base pair coverage to total reads for that specific nucleosome.

To define nucleosome populations from the MNase-seq data, we examined the center of each MNase-seq fragment from the 15-minute time point. Fragments were first filtered by size (107–150 bp), and then the center was determined. All fragment centers were then used to construct histograms (S3 Fig).

### Nucleosome-binding validation assays

Nucleosomal DNA labeled with the fluorescent cyanine dye Cy5 on its 5’ and 3’ ends was formed into purified nucleosomes as described above. Nucleosomes (28 nM) were incubated with increasing amounts of KLF4 (0, 14, 28, 57, 114, 228 nM) in 7 μl DNA-binding buffer on ice for 10 min and then at room temperature for 30 min. KLF4-bound and -unbound nucleosomes were then separated via gel-shift assays using 4%-native-polyacrylamide gels in 0.5 × Tris-borate-EDTA buffer at 100 V at 4 °C. After the gel shift assays, the nucleosomes were visualized and quantified via their Cy5 labels using a ChemiDoc MP imaging system. The intensity of the Cy5 fluorescence was directly proportional to the amount of nucleosomes present, enabling the quantification of the percentage of nucleosome bound.

### DNase-I footprinting

The 186 bp of nucleosomal DNA 5′ labeled with FAM (6-carboxyfluorescein) was formed into nucleosomes and purified as described above. Nucleosomes (50 ng) were bound with 60 nM of KLF4 in DNA binding buffer (10 mM Tris-HCL [pH 7.5], 1 mM MgCl_2_, 10 µM ZnCl_2_, 1 mM DTT, 10 mM KCl, 0.5 mg BSA, 5% glycerol) at room temp for 1 h. Each sample was then incubated with 0.06 U DNase I in 50 µl of digestion buffer (10 mM MgCl_2_, 5 mM ZnCl_2_) at 25 °C for 1 min; digestion was stopped with 90 µl stop solution (NaCl_2_, 30 mM EDTA, 1% SDS). Digested DNA was then purified using phenol/chloroform/isoamyl alcohol and submitted to Roswell Park Genomic Facility for capillary electrophoresis fragment analysis on an ABI PRISM 3130xl Genetic Analyzer. The resulting data were analyzed using the Microsatellite analysis app from Thermo Fisher Scientific.

### Modeling of a KLF4-bound nucleosome

The structure for the Widom 601 nucleosome [73] was retrieved from the Protein Data Bank (PDB) [74] (PDB identifier 5OXV). The location of the KLF4-bound motif within Widom 601 was determined from the Pioneer-seq results, and the motif position was located on the 5OXV nucleosome structure. The relevant nucleotide residues of the motif and the additional footprinted region were colored using the ChimeraX software [75], generating a model of the location and orientation for the motif and the additional footprinted region within the KLF4-bound nucleosome.

## Supporting information

S1 Fig MNase time-course digestion of nucleosome library. Nucleosome library samples were digested with MNase for increasing times (0, 5, 10, 15, 20, and 25 min.) and resolved on a 4% native-PAGE gel stained with SYBR Green. Molecular weight markers (100, 200, 300 bp) are indicated. Digestion over time results in accumulation of protected ~150 bp nucleosomal DNA.
(DOCX)

S2 Fig MNase-seq on nucleosome library. Nucleosome libraries were digested with micrococcal nuclease (MNase) for various times. Sequence reads were then mapped back to a database of the 7500 sequences in the library. Then sequences from the same NPS (601, 5S, MMTV) were pooled together. Mapped fragment ends is used to determine frequency of MNase cleavage at specific bases (left). MNase protection is determined as the ratio of base pair coverage to the total number reads for that specific nucleosome (right). (A) Widom 601 nucleosomes, (B) 5S nucleosomes, (C) MMTV nucleosomes.
(DOCX)

S3 Fig Defining Nucleosome Populations. Histograms for MNase protection centers from 15-minute MNase digestion time points. (A) Widom 601 nucleosomes, (B) 5S nucleosomes, (C) MMTV nucleosomes.
(DOCX)

S4 Fig KLF4 Pioneer-seq binding assays. (A,B,C) 7500 nucleosome sequences were bound to increasing amounts of KLF4 and separated by native PAGE. All assay lanes contain 28 nM nucleosomes with 0, 14, 28, 57, 114 or 228 nM of KLF4. Nucleosome and the supershift (SS) bands are indicated. (D,E,F) Relative supershift for KLF4 binding to the KLF4–1 TFBS (CCCCACCC) at all TF concentrations. (G,H,I) Relative supershift for KLF4 binding to the non-specific TFBS (TGTTTACTTTG) at all TF concentrations.
(DOCX)

S5 Fig Oct4 Pioneer-seq binding assays. (A,B,C) 7500 nucleosome sequences were bound to increasing amounts of OCT4 and separated by native PAGE. All assay lanes contain 28 nM nucleosomes with 0, 14, 28, 57, 114, 228 or 456 nM of OCT4. Nucleosome and the supershift (SS) bands are indicated. (D,E,F) Relative supershift for OCT4 binding to the OCT4–1 TFBS (TATGCAAAT) at all TF concentrations. (G,H,I) Relative supershift for OCT4 binding to the non-specific TFBS (TGTTTACTTTG) at all TF concentrations.
(DOCX)

S6 Fig MYC/MAX Pioneer-seq binding assays. (A,B) 7500 nucleosome sequences were bound to increasing amounts of MYC/MAX and separated by native PAGE. All assay lanes contain 28 nM nucleosomes with 14, 28, 57, 114 or 228 nM of MYC/MAX. Nucleosome and the supershift (SS) bands are indicated. (C,D) Relative supershift for MYC/MAX binding to the Myc-1 TFBS (ACCACGTGGT) at all TF concentrations. (E,F) Relative supershift for MYC/MAX binding to the non-specific TFBS (TGTTTACTTTG) at all TF concentrations.
(DOCX)

S7 Fig Sox2 Pioneer-seq binding assays. (A,B,C) 7500 nucleosome sequences were bound to increasing amounts of SOX2 and separated by native PAGE. All assay lanes contain 28 nM nucleosomes with 0, 14, 28, 57, 114 or 228 nM of SOX2. Nucleosome and the supershift (SS) bands are indicated. (D,E,F) Relative supershift for SOX2 binding to the SOX2–1 TFBS (ACAATGG) at all TF concentrations. (G,H,I) Relative supershift for SOX2 binding to the non-specific TFBS (GGGCATGTCCGGGCATGTCC) at all TF concentrations.
(DOCX)

S8 Fig Binding at linker sites in 601 NPS. Binding of (A) KLF4, (B) MYC, (C) OCT4, and (D) SOX2 to TFBSs located in the left and right linkers of the Widom-601 NPS (that is, TFBSs located outside the 147-bp nucleosome core). For every experiment a non-specific (NS) TFBS is shown for comparison.
(DOCX)

S9 Fig Nucleosome formation efficiency. Nucleosome formation efficiency is determined before nucleosomes are purified from naked DNA by comparing the read numbers for every sequence in the 7500 library to the reads in the naked DNA band.
(DOCX)

S10 Fig KLF4 binding to p53 binding sites. The p53 TFBS are positioned across all possible locations along the (A) Widom-601 nucleosome, (B) 5S nucleosome, or (C) MMTV nucleosome with TFBSs in the left and right linkers to generate a total of 149 unique nucleosomes per TFBS. The relative supershift for each nucleosome is determined by counting the frequency of each sequence within the shifted band in the EMSA and comparing it to that for nonspecific binding. This value is then normalized to the input ratio of nucleosomes (see Eq. 1). Shading around each line is SEM.
(DOCX)

S11 Fig KLF4 binding at a TP53 binding site within a nucleosome. (A) EMSA for KLF4 to four different nucleosomes; Widom-601 control, P53-1 position -21, P53-1 linker, and KLF4–1 linker. Nucleosomes (56 nM) were incubated with increasing amounts of KLF4 (0, 56, 112, 224, 448 nM). EMSA were imaged by staining with SYBR green. (B) DNase-I footprinting of nucleosome containing the p53-A TFBS at position -21. Nucleosome (50 ng) was bound with 120nM of KLF4 in DNA binding buffer.
(DOCX)

S12 Fig Quantify KLF4 binding to p53-1 TFBS. EMSA for KLF4 to four different nucleosomes; Widom-601 control, P53-1 position -32, P53-1 position -21, and P53-1 linker. The concentrations of KLF4 added to each lane were 0, 14, 28, 57, 114, and 228 nM with 28 nM of nucleosome. Binding was quantified from the nucleosome band.
(DOCX)

S13 Fig Binding to *in vivo-*nucleosomes from other studies. The locations of TFBSs with MNase protection for *in vivo*-targeted nucleosomes (ITNs) are shown (red color scale at bottom). MNase protection was measured as the percentage of nucleosome bases that were protected from MNase digestion and calculated for each base pair as the ratio of base-pair coverage to the total reads for that specific nucleosome: (A), NRCAM nucleosome from [31]. (B) ESRRB nucleosome from [61]. (C) ALBN1 nucleosome from [31]. (D) CX3CR1 nucleosome from [31]. The relative supershifts for each nucleosome are shown for KLF4, MYC, OCT4, and SOX2 binding on the right.
(DOCX)

S14 Fig Binding to *in vivo-*targeted nucleosomes with Oct4-Sox2 binding site. (A-D) The locations of TFBSs with MNase protection for *in vivo*-targeted nucleosomes (ITNs) are shown (red color scale at bottom). MNase protection was measured as the percentage of nucleosome bases that were protected from MNase digestion and calculated for each base pair as the ratio of base pair coverage to the total reads for that specific nucleosome.
(DOCX)

S1 Table Information on ITNs. (XLSX)
