# Supplementary material for: Defining transcription factor nucleosome binding with Pioneer-seq
Source: PLoS Genet. 2025 Aug 14;21(8):e1011813. doi: 10.1371/journal.pgen.1011813 (PMC12370185; doi:10.1371/journal.pgen.1011813)
Supplement: S1 Fig — Nucleosome library samples were digested with MNase for increasing times (0, 5, 10, 15, 20, and 25 min.) and resolved on a 4% native-PAGE gel stained with SYBR Green. Molecular weight markers (100, 200, 300 bp) are indicated. Digestion over time results in accumulation of protected ~150 bp nucleosomal DNA. (DOCX) [file pgen.1011813.s001.docx]

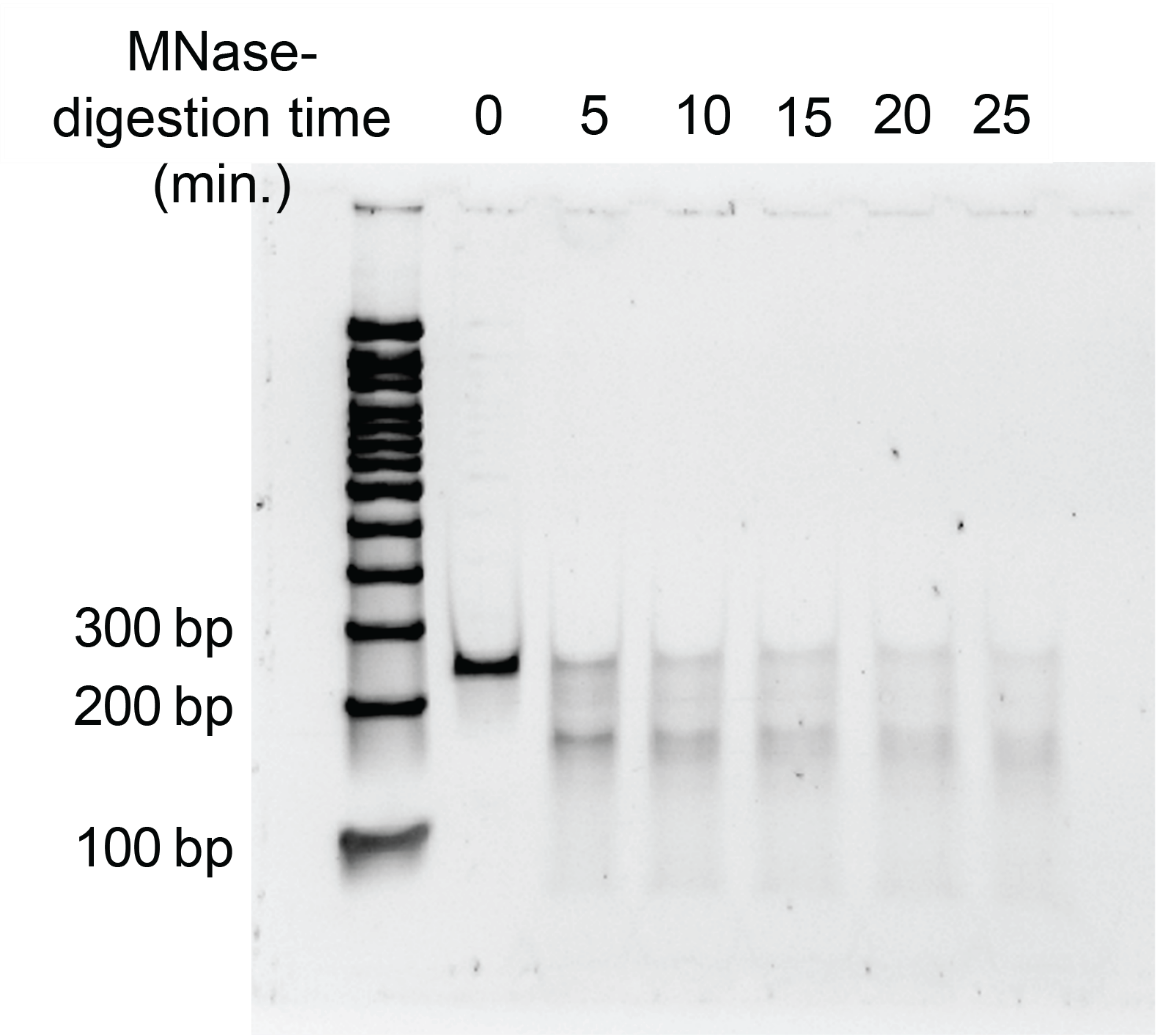


**S1 Fig. MNase time-course digestion of nucleosome library.** Nucleosome library samples were digested with MNase for increasing times (0, 5, 10, 15, 20, and 25 min.) and resolved on a 4% native-PAGE gel stained with SYBR Green. Molecular weight markers (100, 200, 300 bp) are indicated. Digestion over time results in accumulation of protected ~150 bp nucleosomal DNA.
