# Supplementary material for: Defining transcription factor nucleosome binding with Pioneer-seq
Source: PLoS Genet. 2025 Aug 14;21(8):e1011813. doi: 10.1371/journal.pgen.1011813 (PMC12370185; doi:10.1371/journal.pgen.1011813)
Supplement: S2 Fig — Nucleosome libraries were digested with micrococcal nuclease (MNase) for various times. Sequence reads were then mapped back to a database of the 7500 sequences in the library. Then sequences from the same NPS (601, 5S, MMTV) were pooled together. Mapped fragment ends is used to determine frequency of MNase cleavage at specific bases (left). MNase protection is determined as the ratio of base pair coverage to the total number reads for that specific nucleosome (right). (A) Widom 601 nucleosomes, (B) 5S nucleosomes, (C) MMTV nucleosomes. (DOCX) [file pgen.1011813.s002.docx]

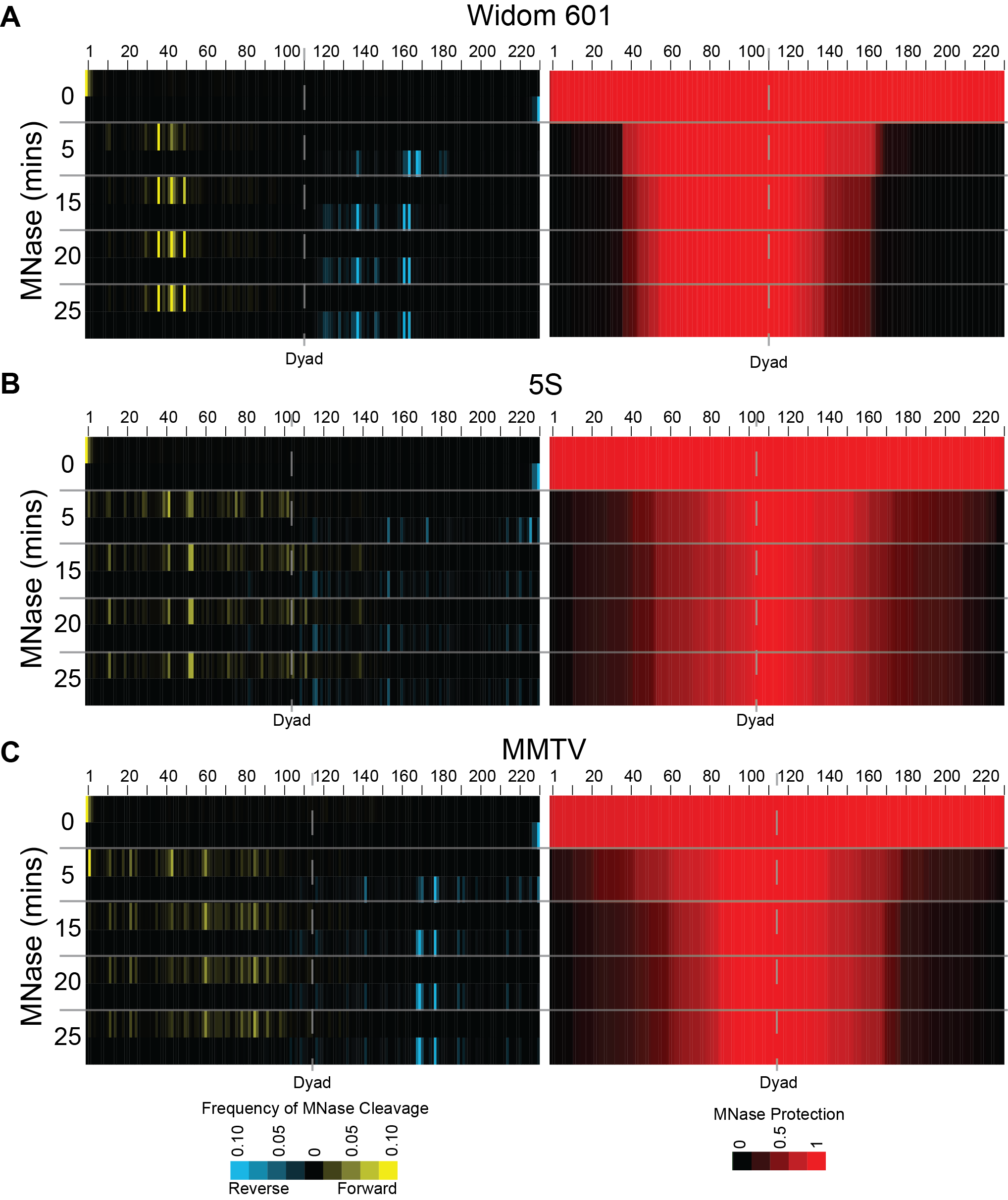


**S2 Fig. MNase-seq on nucleosome library.** Nucleosome libraries were digested with micrococcal nuclease (MNase) for various times. Sequence reads were then mapped back to a database of the 7500 sequences in the library. Then sequences from the same NPS (601, 5S, MMTV) were pooled together. Mapped fragment ends is used to determine frequency of MNase cleavage at specific bases (left). MNase protection is determined as the ratio of base pair coverage to the total number reads for that specific nucleosome (right). **(A)** Widom 601 nucleosomes, **(B)** 5S nucleosomes, **(C)** MMTV nucleosomes.
