# Supplementary material for: Defining transcription factor nucleosome binding with Pioneer-seq
Source: PLoS Genet. 2025 Aug 14;21(8):e1011813. doi: 10.1371/journal.pgen.1011813 (PMC12370185; doi:10.1371/journal.pgen.1011813)
Supplement: S3 Fig — Histograms for MNase protection centers from 15-minute MNase digestion time points. (A) Widom 601 nucleosomes, (B) 5S nucleosomes, (C) MMTV nucleosomes. (DOCX) [file pgen.1011813.s003.docx]

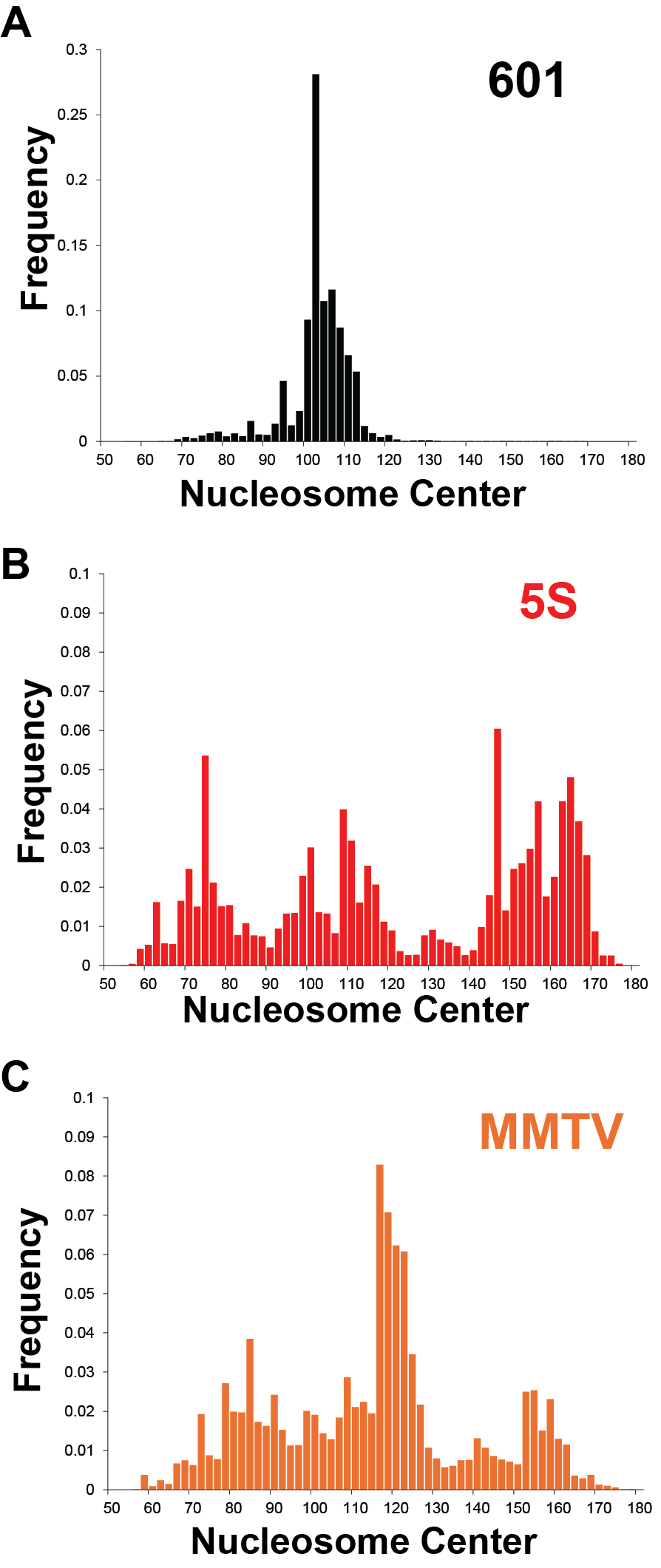


**S3 Fig. Defining Nucleosome Populations.** Histograms for MNase protection centers from 15-minute MNase digestion time points. **(A)** Widom 601 nucleosomes, **(B)** 5S nucleosomes, **(C)** MMTV nucleosomes.
