# Supplementary material for: Defining transcription factor nucleosome binding with Pioneer-seq
Source: PLoS Genet. 2025 Aug 14;21(8):e1011813. doi: 10.1371/journal.pgen.1011813 (PMC12370185; doi:10.1371/journal.pgen.1011813)
Supplement: S5 Fig — (A,B,C) 7500 nucleosome sequences were bound to increasing amounts of OCT4 and separated by native PAGE. All assay lanes contain 28 nM nucleosomes with 0, 14, 28, 57, 114, 228 or 456 nM of OCT4. Nucleosome and the supershift (SS) bands are indicated. (D,E,F) Relative supershift for OCT4 binding to the OCT4–1 TFBS (TATGCAAAT) at all TF concentrations. (G,H,I) Relative supershift for OCT4 binding to the non-specific TFBS (TGTTTACTTTG) at all TF concentrations. (DOCX) [file pgen.1011813.s005.docx]

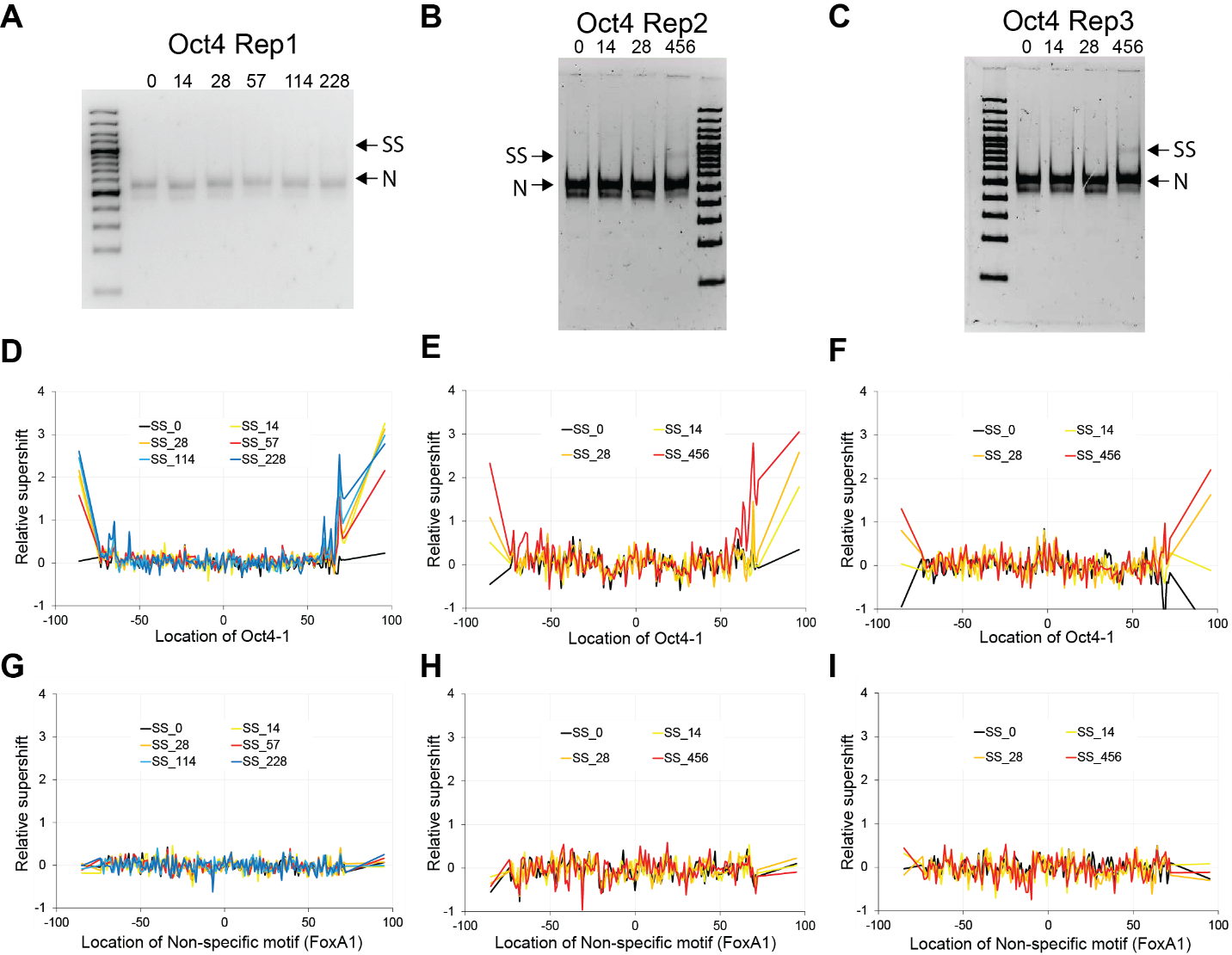


**S5 Fig. Oct4 Pioneer-seq binding assays.** (**A**,**B**,**C**) 7500 nucleosome sequences were bound to increasing amounts of OCT4 and separated by native PAGE. All assay lanes contain 28 nM nucleosomes with 0, 14, 28, 57, 114, 228 or 456 nM of OCT4. Nucleosome and the supershift (SS) bands are indicated. (**D,E,F**) Relative supershift for OCT4 binding to the OCT4-1 TFBS (TATGCAAAT) at all TF concentrations. (**G,H,I**) Relative supershift for OCT4 binding to the non-specific TFBS (TGTTTACTTTG) at all TF concentrations.
