# Supplementary material for: Defining transcription factor nucleosome binding with Pioneer-seq
Source: PLoS Genet. 2025 Aug 14;21(8):e1011813. doi: 10.1371/journal.pgen.1011813 (PMC12370185; doi:10.1371/journal.pgen.1011813)
Supplement: S6 Fig — (A,B) 7500 nucleosome sequences were bound to increasing amounts of MYC/MAX and separated by native PAGE. All assay lanes contain 28 nM nucleosomes with 14, 28, 57, 114 or 228 nM of MYC/MAX. Nucleosome and the supershift (SS) bands are indicated. (C,D) Relative supershift for MYC/MAX binding to the Myc-1 TFBS (ACCACGTGGT) at all TF concentrations. (E,F) Relative supershift for MYC/MAX binding to the non-specific TFBS (TGTTTACTTTG) at all TF concentrations. (DOCX) [file pgen.1011813.s006.docx]

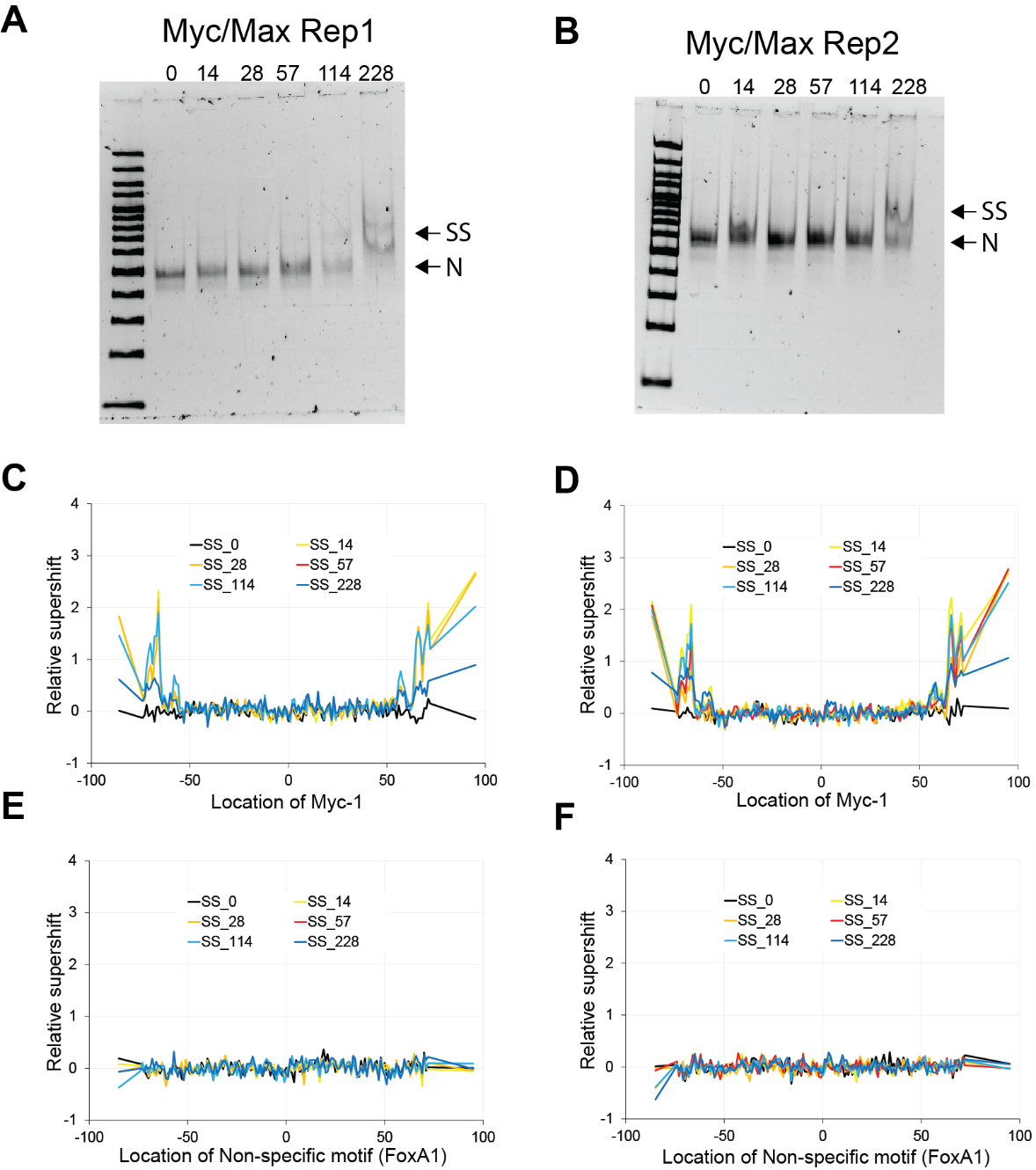


**S6 Fig. MYC/MAX Pioneer-seq binding assays.** (**A**,**B**) 7500 nucleosome sequences were bound to increasing amounts of MYC/MAX and separated by native PAGE. All assay lanes contain 28 nM nucleosomes with 14, 28, 57, 114 or 228 nM of MYC/MAX. Nucleosome and the supershift (SS) bands are indicated. (**C,D**) Relative supershift for MYC/MAX binding to the Myc-1 TFBS (ACCACGTGGT) at all TF concentrations. (**E,F**) Relative supershift for MYC/MAX binding to the non-specific TFBS (TGTTTACTTTG) at all TF concentrations.
