# Supplementary material for: Defining transcription factor nucleosome binding with Pioneer-seq
Source: PLoS Genet. 2025 Aug 14;21(8):e1011813. doi: 10.1371/journal.pgen.1011813 (PMC12370185; doi:10.1371/journal.pgen.1011813)
Supplement: S7 Fig — (A,B,C) 7500 nucleosome sequences were bound to increasing amounts of SOX2 and separated by native PAGE. All assay lanes contain 28 nM nucleosomes with 0, 14, 28, 57, 114 or 228 nM of SOX2. Nucleosome and the supershift (SS) bands are indicated. (D,E,F) Relative supershift for SOX2 binding to the SOX2–1 TFBS (ACAATGG) at all TF concentrations. (G,H,I) Relative supershift for SOX2 binding to the non-specific TFBS (GGGCATGTCCGGGCATGTCC) at all TF concentrations. (DOCX) [file pgen.1011813.s007.docx]

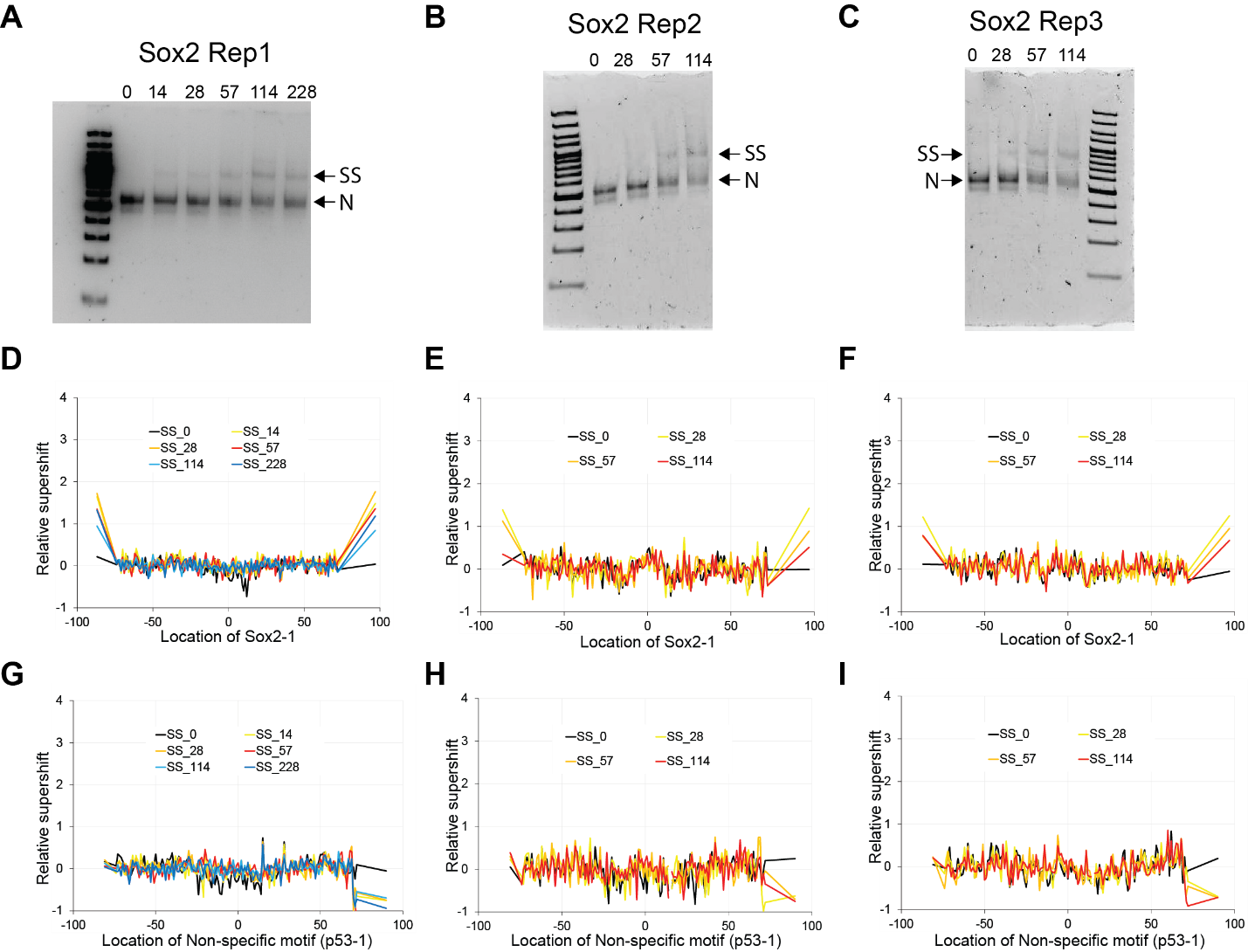


**S7 Fig. Sox2 Pioneer-seq binding assays.** (**A**,**B**,**C**) 7500 nucleosome sequences were bound to increasing amounts of SOX2 and separated by native PAGE. All assay lanes contain 28 nM nucleosomes with 0, 14, 28, 57, 114 or 228 nM of SOX2. Nucleosome and the supershift (SS) bands are indicated. (**D,E,F**) Relative supershift for SOX2 binding to the SOX2-1 TFBS (ACAATGG) at all TF concentrations. (**G,H,I**) Relative supershift for SOX2 binding to the non-specific TFBS (GGGCATGTCCGGGCATGTCC) at all TF concentrations.
