# Supplementary material for: Defining transcription factor nucleosome binding with Pioneer-seq
Source: PLoS Genet. 2025 Aug 14;21(8):e1011813. doi: 10.1371/journal.pgen.1011813 (PMC12370185; doi:10.1371/journal.pgen.1011813)
Supplement: S8 Fig — Binding of (A) KLF4, (B) MYC, (C) OCT4, and (D) SOX2 to TFBSs located in the left and right linkers of the Widom-601 NPS (that is, TFBSs located outside the 147-bp nucleosome core). For every experiment a non-specific (NS) TFBS is shown for comparison. (DOCX) [file pgen.1011813.s008.docx]

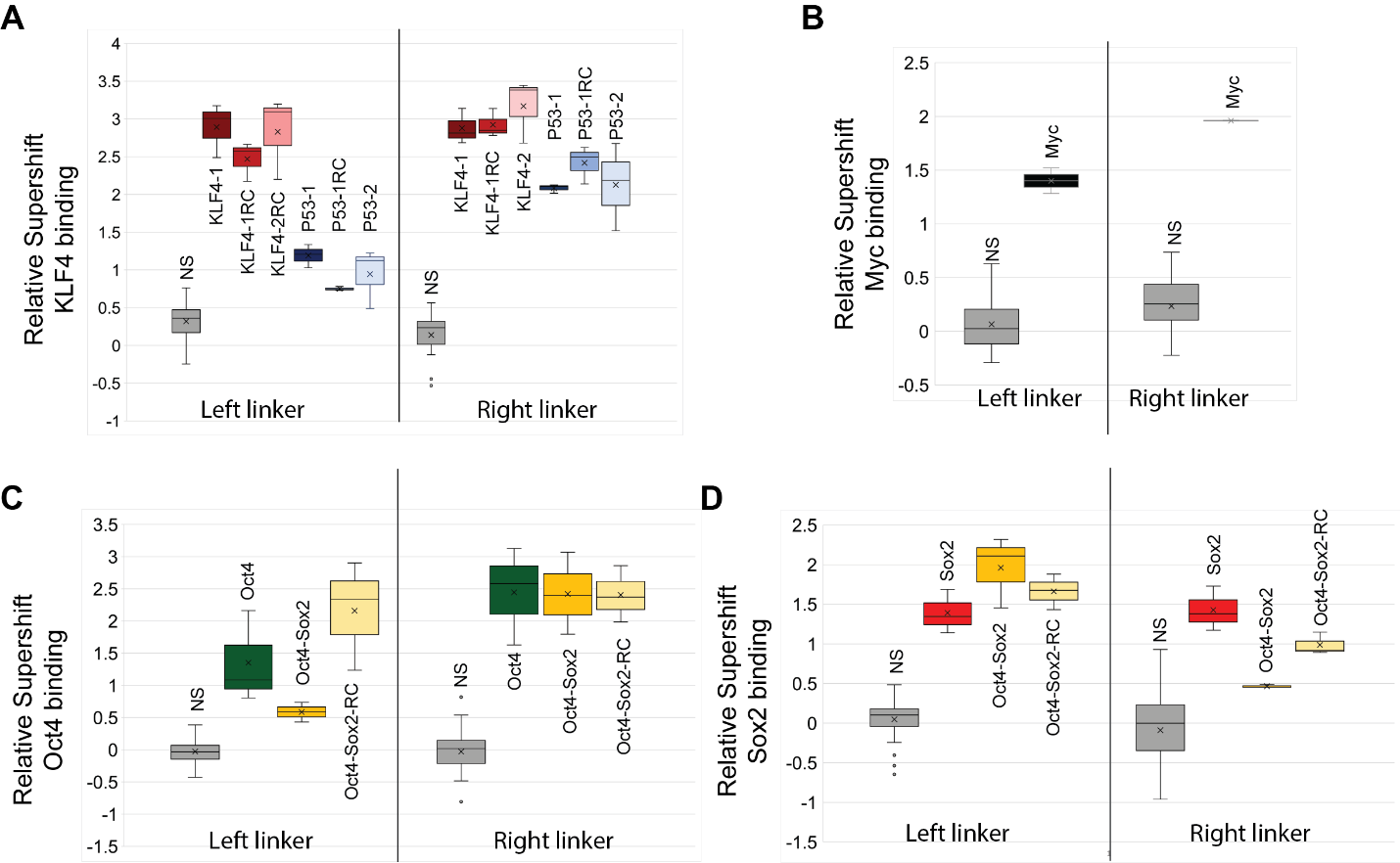


**S8 Fig.** **Binding at linker sites in 601 NPS.** Binding of **(A)** KLF4, **(B)** MYC, **(C)** OCT4, and **(D)** SOX2 to TFBSs located in the left and right linkers of the Widom-601 NPS (that is, TFBSs located outside the 147-bp nucleosome core). For every experiment a non-specific (NS) TFBS is shown for comparison.
