# Supplementary material for: Defining transcription factor nucleosome binding with Pioneer-seq
Source: PLoS Genet. 2025 Aug 14;21(8):e1011813. doi: 10.1371/journal.pgen.1011813 (PMC12370185; doi:10.1371/journal.pgen.1011813)
Supplement: S9 Fig — Nucleosome formation efficiency is determined before nucleosomes are purified from naked DNA by comparing the read numbers for every sequence in the 7500 library to the reads in the naked DNA band. (DOCX) [file pgen.1011813.s009.docx]

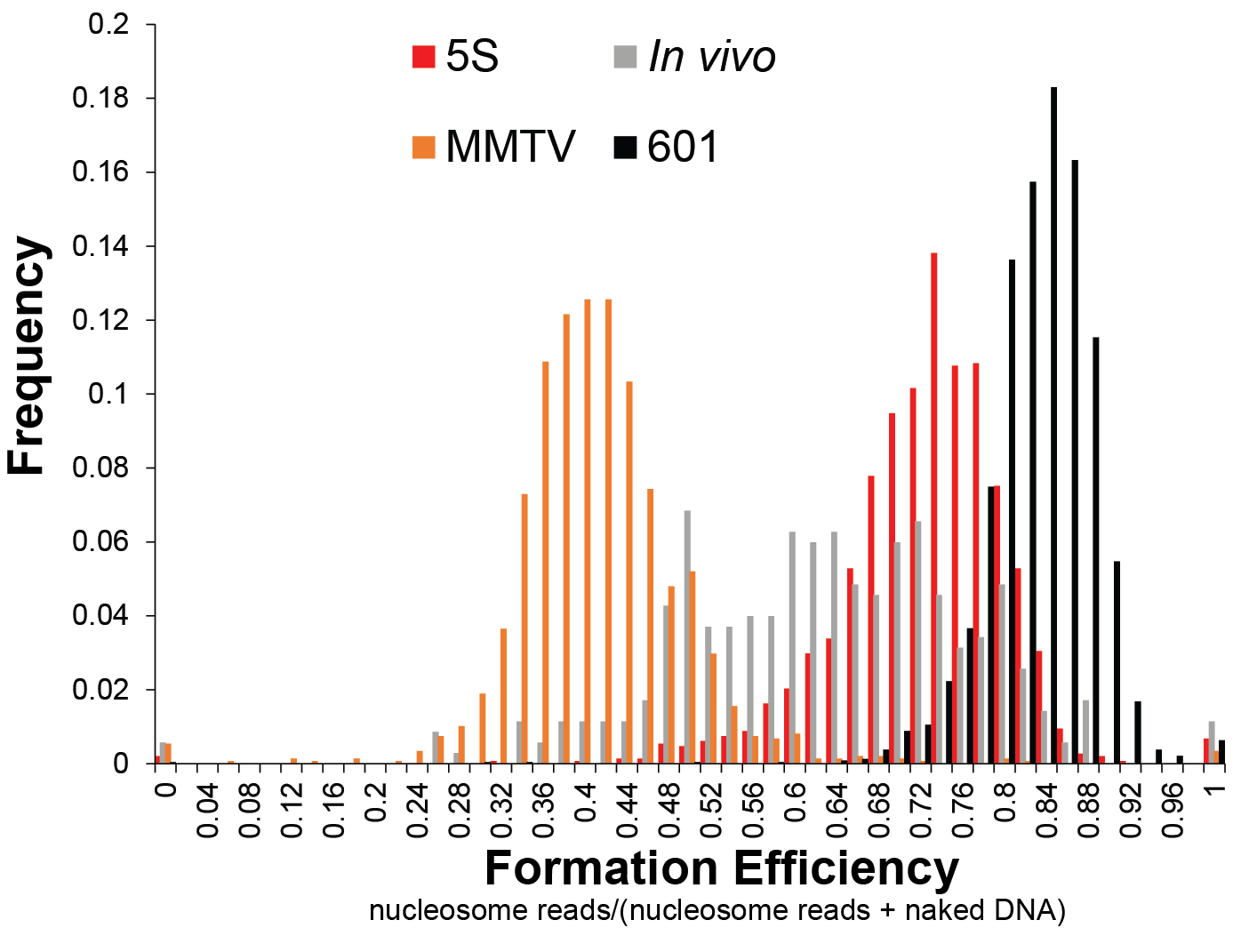


**S9 Fig.** **Nucleosome formation efficiency.** Nucleosome formation efficiency is determined before nucleosomes are purified from naked DNA by comparing the read numbers for every sequence in the 7500 library to the reads in the naked DNA band.
