# Supplementary material for: Defining transcription factor nucleosome binding with Pioneer-seq
Source: PLoS Genet. 2025 Aug 14;21(8):e1011813. doi: 10.1371/journal.pgen.1011813 (PMC12370185; doi:10.1371/journal.pgen.1011813)
Supplement: S10 Fig — The p53 TFBS are positioned across all possible locations along the (A) Widom-601 nucleosome, (B) 5S nucleosome, or (C) MMTV nucleosome with TFBSs in the left and right linkers to generate a total of 149 unique nucleosomes per TFBS. The relative supershift for each nucleosome is determined by counting the frequency of each sequence within the shifted band in the EMSA and comparing it to that for nonspecific binding. This value is then normalized to the input ratio of nucleosomes (see Eq. 1). Shading around each line is SEM. (DOCX) [file pgen.1011813.s010.docx]

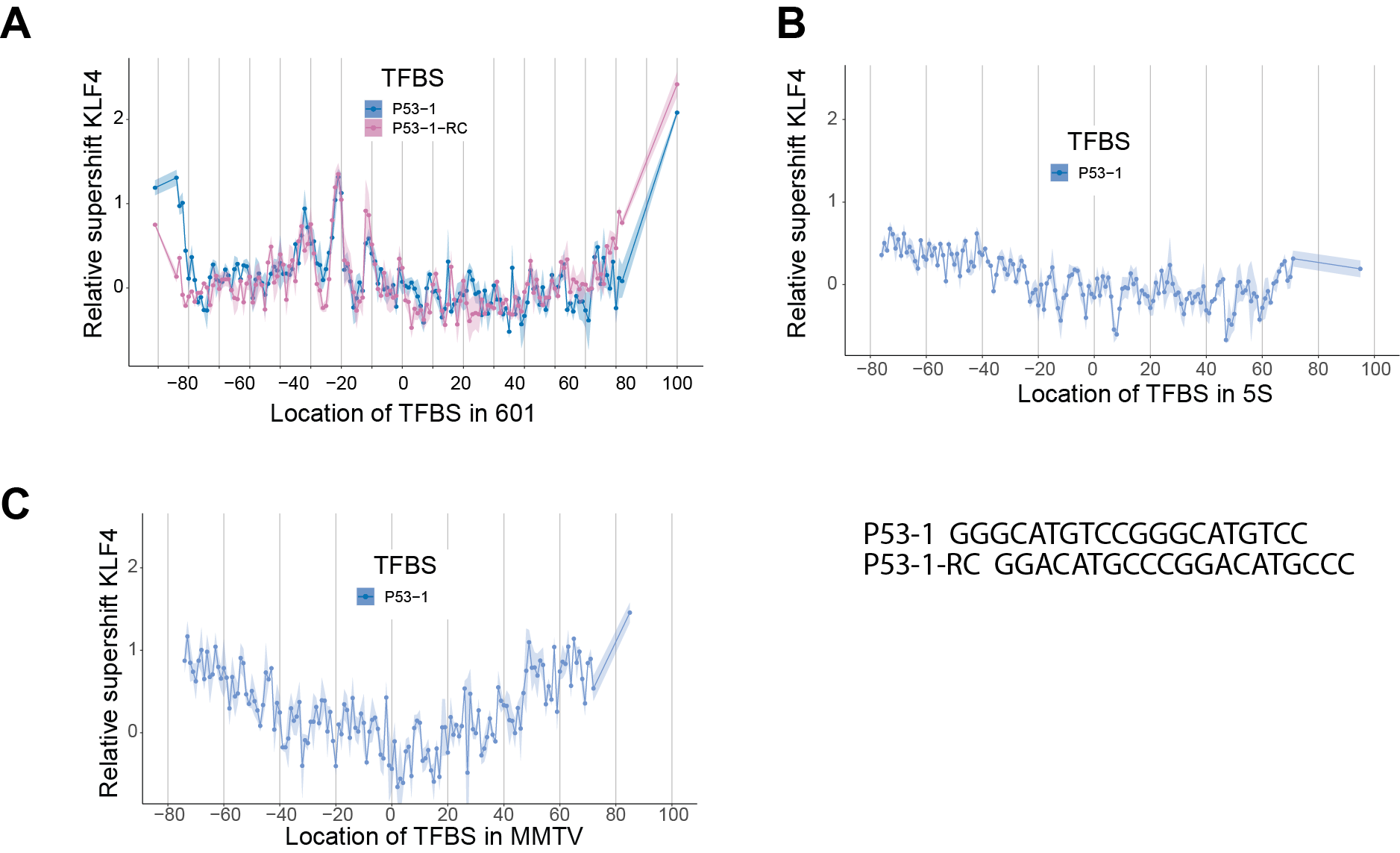


**S10 Fig.** **KLF4 binding to p53 binding sites.** The p53 TFBS are positioned across all possible locations along the (**A**) Widom-601 nucleosome, (**B**) 5S nucleosome, or (**C**) MMTV nucleosome with TFBSs in the left and right linkers to generate a total of 149 unique nucleosomes per TFBS. The relative supershift for each nucleosome is determined by counting the frequency of each sequence within the shifted band in the EMSA and comparing it to that for nonspecific binding. This value is then normalized to the input ratio of nucleosomes (see Eq. 1). Shading around each line is SEM.
