# Supplementary material for: Defining transcription factor nucleosome binding with Pioneer-seq
Source: PLoS Genet. 2025 Aug 14;21(8):e1011813. doi: 10.1371/journal.pgen.1011813 (PMC12370185; doi:10.1371/journal.pgen.1011813)
Supplement: S11 Fig — (A) EMSA for KLF4 to four different nucleosomes; Widom-601 control, P53-1 position -21, P53-1 linker, and KLF4–1 linker. Nucleosomes (56 nM) were incubated with increasing amounts of KLF4 (0, 56, 112, 224, 448 nM). EMSA were imaged by staining with SYBR green. (B) DNase-I footprinting of nucleosome containing the p53-A TFBS at position -21. Nucleosome (50 ng) was bound with 120nM of KLF4 in DNA binding buffer. (DOCX) [file pgen.1011813.s011.docx]

**
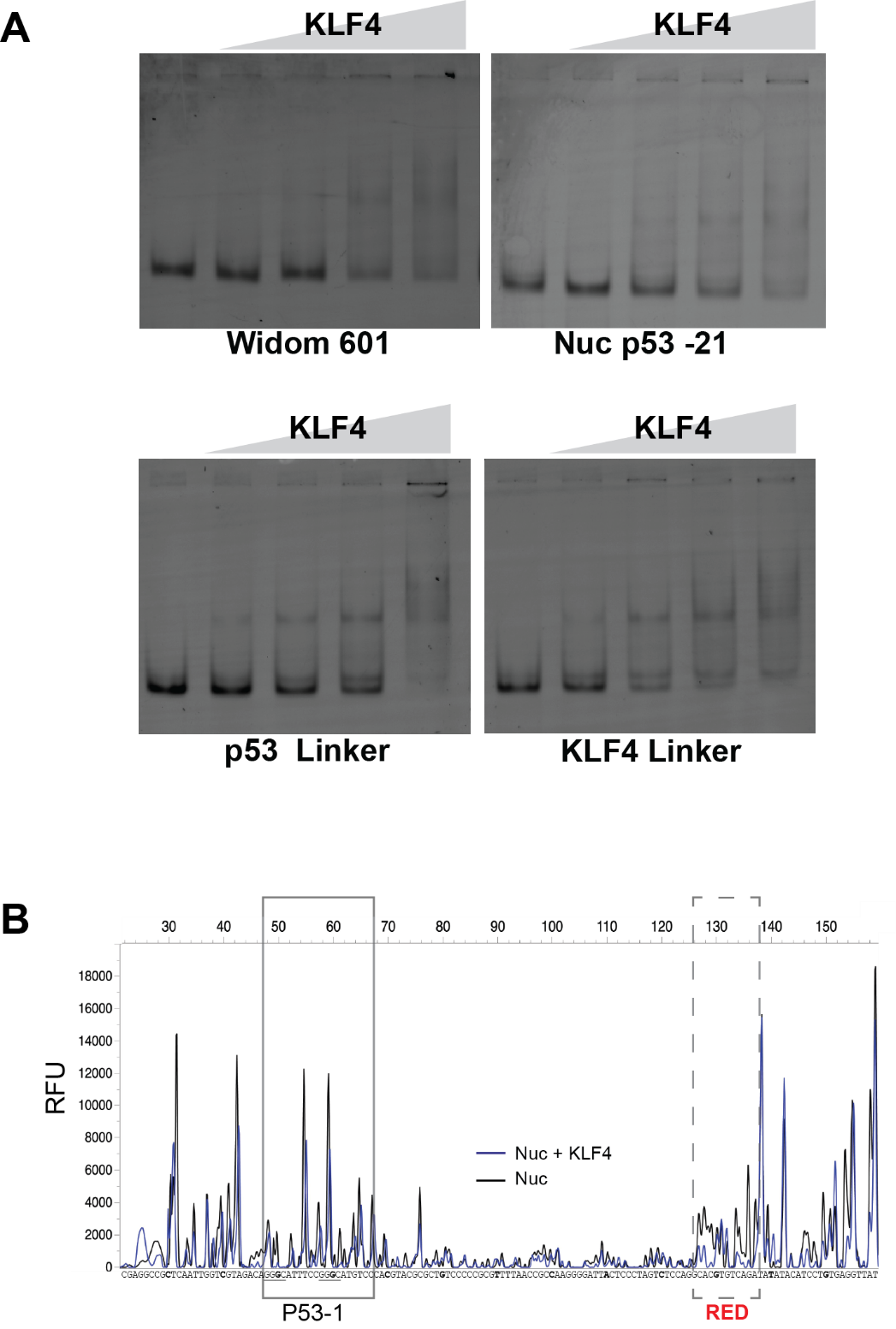
**

**S11 Fig. KLF4 binding at a TP53 binding site within a nucleosome. (A)** EMSA for KLF4 to four different nucleosomes; Widom-601 control, P53-1 position -21, P53-1 linker, and KLF4-1 linker. Nucleosomes (56 nM) were incubated with increasing amounts of KLF4 (0, 56, 112, 224, 448 nM). EMSA were imaged by staining with SYBR green. **(B)** DNase-I footprinting of nucleosome containing the p53-A TFBS at position -21. Nucleosome (50 ng) was bound with 120nM of KLF4 in DNA binding buffer.
