# Supplementary material for: Defining transcription factor nucleosome binding with Pioneer-seq
Source: PLoS Genet. 2025 Aug 14;21(8):e1011813. doi: 10.1371/journal.pgen.1011813 (PMC12370185; doi:10.1371/journal.pgen.1011813)
Supplement: S12 Fig — EMSA for KLF4 to four different nucleosomes; Widom-601 control, P53-1 position -32, P53-1 position -21, and P53-1 linker. The concentrations of KLF4 added to each lane were 0, 14, 28, 57, 114, and 228 nM with 28 nM of nucleosome. Binding was quantified from the nucleosome band. (DOCX) [file pgen.1011813.s012.docx]

**
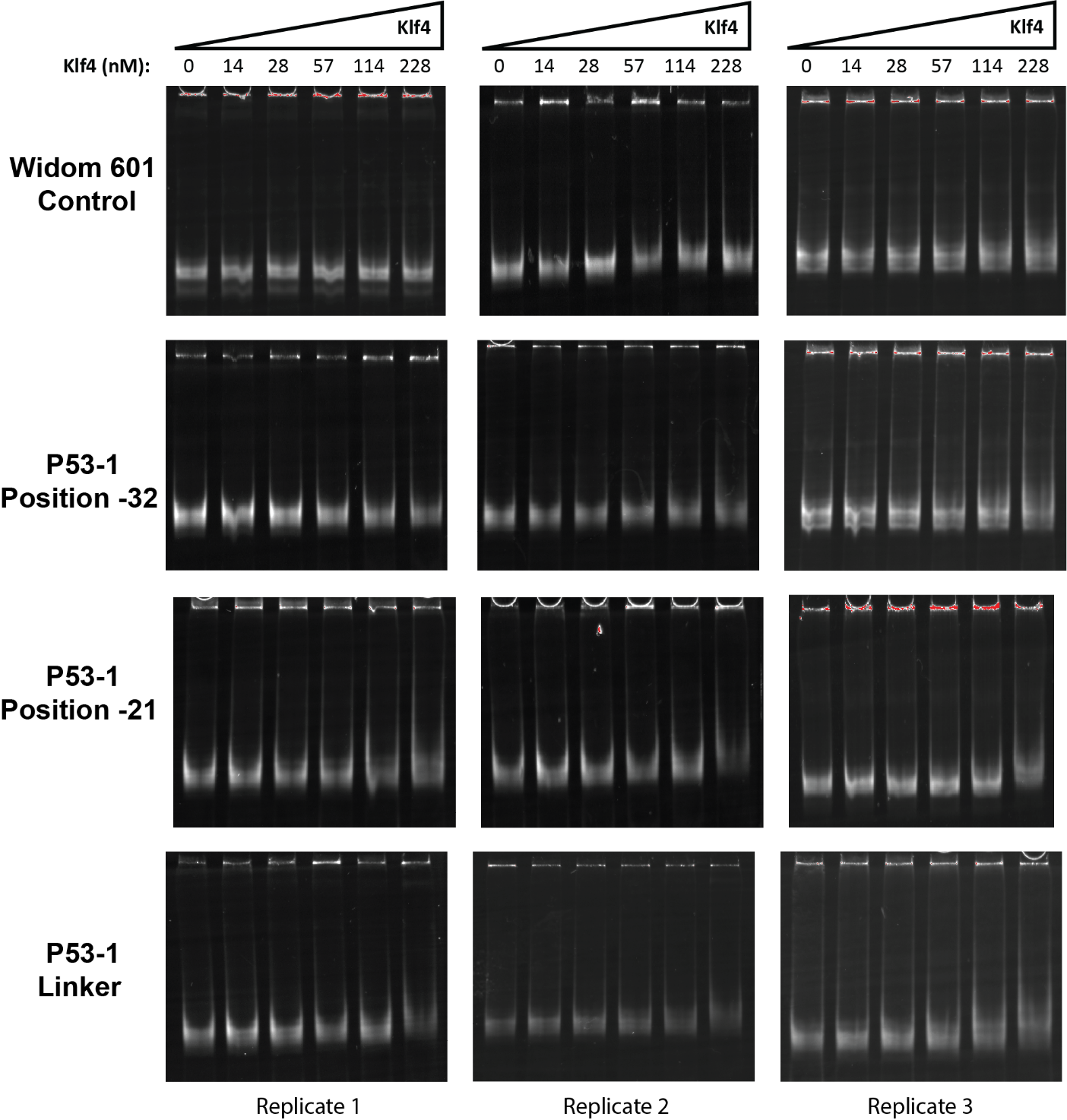
**

**S12 Fig.** **Quantify** **KLF4 binding to p53-1 TFBS.** EMSA for KLF4 to four different nucleosomes; Widom-601 control, P53-1 position -32, P53-1 position -21, and P53-1 linker. The concentrations of KLF4 added to each lane were 0, 14, 28, 57, 114, and 228 nM with 28 nM of nucleosome. Binding was quantified from the nucleosome band.
