# Supplementary material for: Defining transcription factor nucleosome binding with Pioneer-seq
Source: PLoS Genet. 2025 Aug 14;21(8):e1011813. doi: 10.1371/journal.pgen.1011813 (PMC12370185; doi:10.1371/journal.pgen.1011813)
Supplement: S13 Fig — The locations of TFBSs with MNase protection for in vivo-targeted nucleosomes (ITNs) are shown (red color scale at bottom). MNase protection was measured as the percentage of nucleosome bases that were protected from MNase digestion and calculated for each base pair as the ratio of base-pair coverage to the total reads for that specific nucleosome: (A), NRCAM nucleosome from [31]. (B) ESRRB nucleosome from [61]. (C) ALBN1 nucleosome from [31]. (D) CX3CR1 nucleosome from [31]. The relative supershifts for each nucleosome are shown for KLF4, MYC, OCT4, and SOX2 binding on the right. (DOCX) [file pgen.1011813.s013.docx]

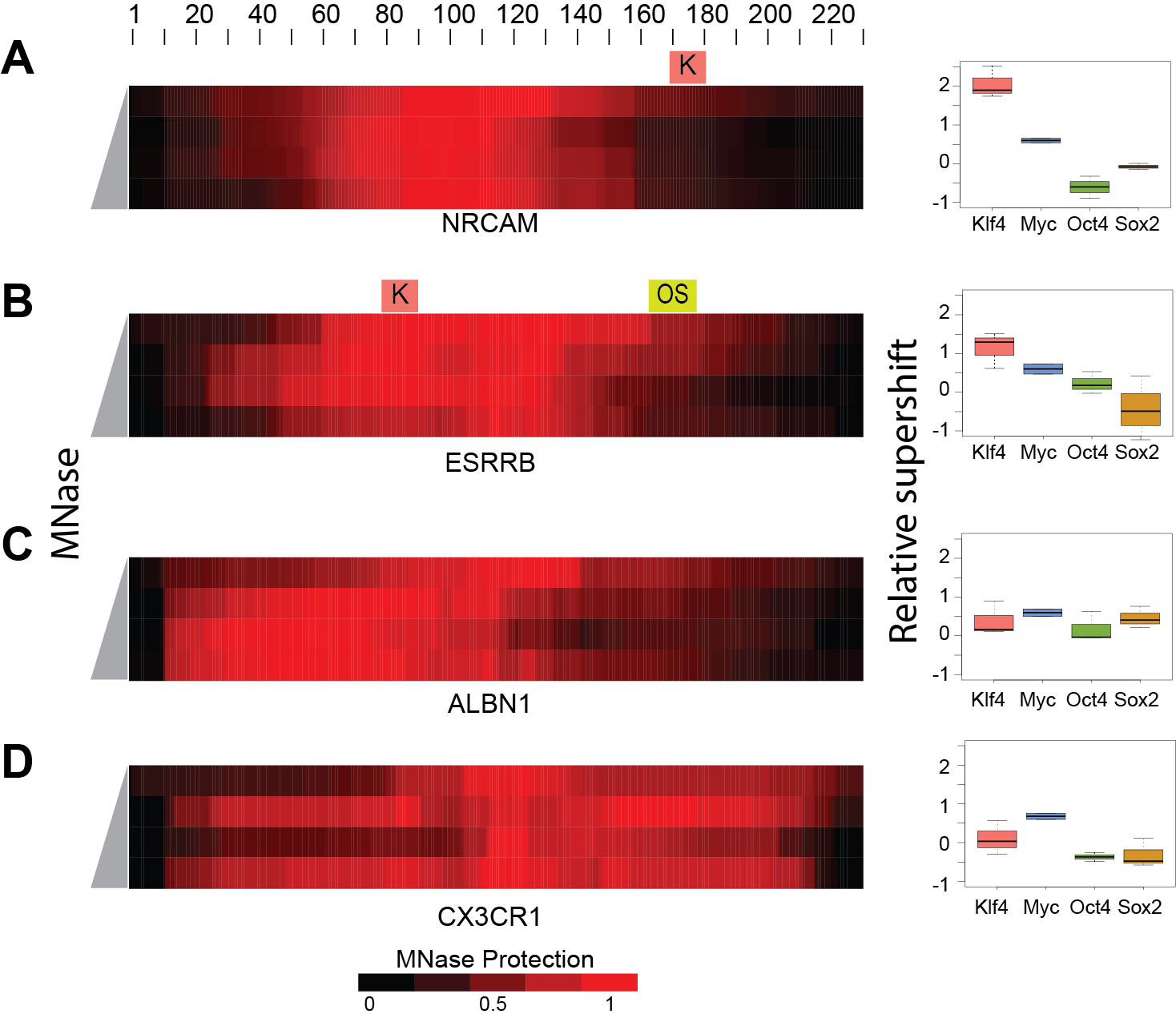


**S13 Fig.** **Binding to *in vivo-*nucleosomes from other studies.** The locations of TFBSs with MNase protection for *in vivo*-targeted nucleosomes (ITNs) are shown (red color scale at bottom). MNase protection was measured as the percentage of nucleosome bases that were protected from MNase digestion and calculated for each base pair as the ratio of base-pair coverage to the total reads for that specific nucleosome: **(A)**, NRCAM nucleosome from Garcia *et al* 2019. (**B)** ESRRB nucleosome from Huertas *et al* 2020. (**C)** ALBN1 nucleosome from Garcia *et al* 2019. (**D**) CX3CR1 nucleosome from Garcia *et al* 2019. The relative supershifts for each nucleosome are shown for KLF4, MYC, OCT4, and SOX2 binding on the right.
