# Supplementary material for: Defining transcription factor nucleosome binding with Pioneer-seq
Source: PLoS Genet. 2025 Aug 14;21(8):e1011813. doi: 10.1371/journal.pgen.1011813 (PMC12370185; doi:10.1371/journal.pgen.1011813)
Supplement: S14 Fig — (A-D) The locations of TFBSs with MNase protection for in vivo-targeted nucleosomes (ITNs) are shown (red color scale at bottom). MNase protection was measured as the percentage of nucleosome bases that were protected from MNase digestion and calculated for each base pair as the ratio of base pair coverage to the total reads for that specific nucleosome. (DOCX) [file pgen.1011813.s014.docx]

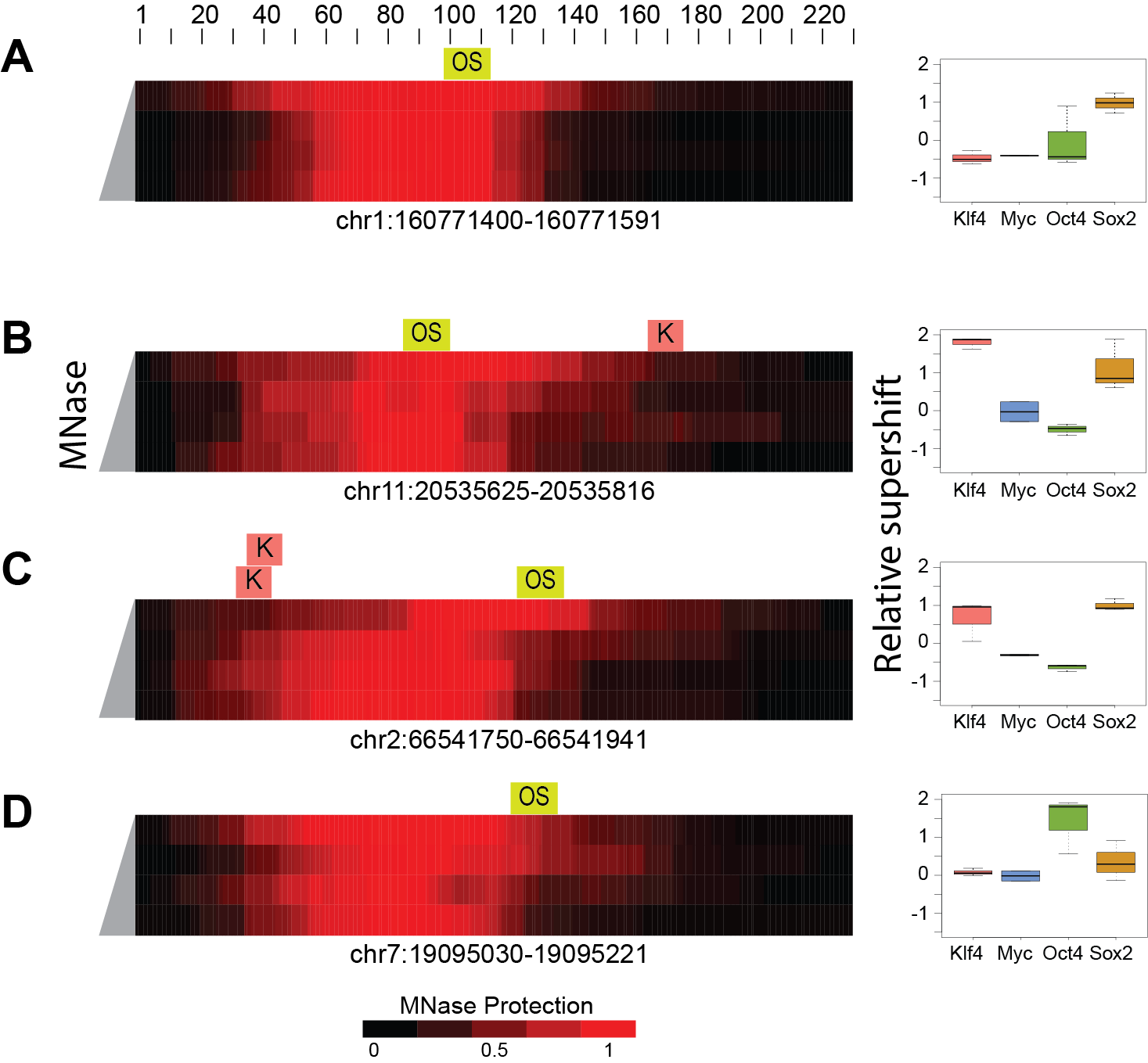


**S14 Fig.** **Binding to *in vivo-*targeted nucleosomes with Oct4-Sox2 binding site. (A-D)** The locations of TFBSs with MNase protection for *in vivo*-targeted nucleosomes (ITNs) are shown (red color scale at bottom). MNase protection was measured as the percentage of nucleosome bases that were protected from MNase digestion and calculated for each base pair as the ratio of base pair coverage to the total reads for that specific nucleosome.
